# Search for light Higgs bosons from supersymmetric cascade decays in $\text {pp}$ collisions at $\sqrt{s}=13\,\textrm{TeV}$

**DOI:** 10.1140/epjc/s10052-023-11581-0

**Published:** 2023-07-06

**Authors:** A. Tumasyan, W. Adam, J. W. Andrejkovic, T. Bergauer, S. Chatterjee, K. Damanakis, M. Dragicevic, A. Escalante Del Valle, R. Frühwirth, M. Jeitler, N. Krammer, L. Lechner, D. Liko, I. Mikulec, P. Paulitsch, F. M. Pitters, J. Schieck, R. Schöfbeck, D. Schwarz, S. Templ, W. Waltenberger, C.-E. Wulz, M. R. Darwish, E. A. De Wolf, T. Janssen, T. Kello, A. Lelek, H. Rejeb Sfar, P. Van Mechelen, S. Van Putte, N. Van Remortel, E. S. Bols, J. D’Hondt, A. De Moor, M. Delcourt, H. El Faham, S. Lowette, S. Moortgat, A. Morton, D. Müller, A. R. Sahasransu, S. Tavernier, W. Van Doninck, D. Vannerom, D. Beghin, B. Bilin, B. Clerbaux, G. De Lentdecker, L. Favart, A. K. Kalsi, K. Lee, M. Mahdavikhorrami, I. Makarenko, L. Moureaux, S. Paredes, L. Pétré, A. Popov, N. Postiau, E. Starling, L. Thomas, M. Vanden Bemden, C. Vander Velde, P. Vanlaer, T. Cornelis, D. Dobur, J. Knolle, L. Lambrecht, G. Mestdach, M. Niedziela, C. Rendón, C. Roskas, A. Samalan, K. Skovpen, M. Tytgat, B. Vermassen, L. Wezenbeek, A. Benecke, A. Bethani, G. Bruno, F. Bury, C. Caputo, P. David, C. Delaere, I. S. Donertas, A. Giammanco, K. Jaffel, Sa. Jain, V. Lemaitre, K. Mondal, J. Prisciandaro, A. Taliercio, M. Teklishyn, T. T. Tran, P. Vischia, S. Wertz, G. A. Alves, C. Hensel, A. Moraes, P. Rebello Teles, W. L. Aldá Júnior, M. Alves Gallo Pereira, M. Barroso Ferreira Filho, H. Brandao Malbouisson, W. Carvalho, J. Chinellato, E. M. Da Costa, G. G. Da Silveira, D. De Jesus Damiao, V. Dos Santos Sousa, S. Fonseca De Souza, C. Mora Herrera, K. Mota Amarilo, L. Mundim, H. Nogima, A. Santoro, S. M. Silva Do Amaral, A. Sznajder, M. Thiel, F. Torres Da Silva De Araujo, A. Vilela Pereira, C. A. Bernardes, L. Calligaris, T. R. Fernandez Perez Tomei, E. M. Gregores, D. S. Lemos, P. G. Mercadante, S. F. Novaes, Sandra S. Padula, A. Aleksandrov, G. Antchev, R. Hadjiiska, P. Iaydjiev, M. Misheva, M. Rodozov, M. Shopova, G. Sultanov, A. Dimitrov, T. Ivanov, L. Litov, B. Pavlov, P. Petkov, A. Petrov, T. Cheng, T. Javaid, M. Mittal, L. Yuan, M. Ahmad, G. Bauer, C. Dozen, Z. Hu, J. Martins, Y. Wang, K. Yi, E. Chapon, G. M. Chen, H. S. Chen, M. Chen, F. Iemmi, A. Kapoor, D. Leggat, H. Liao, Z.-A. Liu, V. Milosevic, F. Monti, R. Sharma, J. Tao, J. Thomas-Wilsker, J. Wang, H. Zhang, J. Zhao, A. Agapitos, Y. An, Y. Ban, C. Chen, A. Levin, Q. Li, X. Lyu, Y. Mao, S. J. Qian, D. Wang, J. Xiao, H. Yang, M. Lu, Z. You, X. Gao, H. Okawa, Y. Zhang, Z. Lin, M. Xiao, C. Avila, A. Cabrera, C. Florez, J. Fraga, J. Mejia Guisao, F. Ramirez, J. D. Ruiz Alvarez, D. Giljanovic, N. Godinovic, D. Lelas, I. Puljak, Z. Antunovic, M. Kovac, T. Sculac, V. Brigljevic, D. Ferencek, D. Majumder, M. Roguljic, A. Starodumov, T. Susa, A. Attikis, K. Christoforou, A. Ioannou, G. Kole, M. Kolosova, S. Konstantinou, J. Mousa, C. Nicolaou, F. Ptochos, P. A. Razis, H. Rykaczewski, H. Saka, M. Finger, M. Finger, A. Kveton, E. Ayala, E. Carrera Jarrin, A. A. Abdelalim, S. Elgammal, M. A. Mahmoud, Y. Mohammed, S. Bhowmik, R. K. Dewanjee, K. Ehataht, M. Kadastik, S. Nandan, C. Nielsen, J. Pata, M. Raidal, L. Tani, C. Veelken, P. Eerola, H. Kirschenmann, K. Osterberg, M. Voutilainen, S. Bharthuar, E. Brücken, F. Garcia, J. Havukainen, M. S. Kim, R. Kinnunen, T. Lampén, K. Lassila-Perini, S. Lehti, T. Lindén, M. Lotti, L. Martikainen, M. Myllymäki, J. Ott, H. Siikonen, E. Tuominen, J. Tuominiemi, P. Luukka, H. Petrow, T. Tuuva, C. Amendola, M. Besancon, F. Couderc, M. Dejardin, D. Denegri, J. L. Faure, F. Ferri, S. Ganjour, P. Gras, G. Hamel de Monchenault, P. Jarry, B. Lenzi, E. Locci, J. Malcles, J. Rander, A. Rosowsky, M.Ö. Sahin, A. Savoy-Navarro, M. Titov, G. B. Yu, S. Ahuja, F. Beaudette, M. Bonanomi, A. Buchot Perraguin, P. Busson, A. Cappati, C. Charlot, O. Davignon, B. Diab, G. Falmagne, S. Ghosh, R. Granier de Cassagnac, A. Hakimi, I. Kucher, J. Motta, M. Nguyen, C. Ochando, P. Paganini, J. Rembser, R. Salerno, U. Sarkar, J. B. Sauvan, Y. Sirois, A. Tarabini, A. Zabi, A. Zghiche, J.-L. Agram, J. Andrea, D. Apparu, D. Bloch, G. Bourgatte, J.-M. Brom, E. C. Chabert, C. Collard, D. Darej, J.-C. Fontaine, U. Goerlach, C. Grimault, A.-C. Le Bihan, E. Nibigira, P. Van Hove, E. Asilar, S. Beauceron, C. Bernet, G. Boudoul, C. Camen, A. Carle, N. Chanon, D. Contardo, P. Depasse, H. El Mamouni, J. Fay, S. Gascon, M. Gouzevitch, B. Ille, I. B. Laktineh, H. Lattaud, A. Lesauvage, M. Lethuillier, L. Mirabito, S. Perries, K. Shchablo, V. Sordini, L. Torterotot, G. Touquet, M. Vander Donckt, S. Viret, I. Bagaturia, I. Lomidze, Z. Tsamalaidze, V. Botta, L. Feld, K. Klein, M. Lipinski, D. Meuser, A. Pauls, N. Röwert, J. Schulz, M. Teroerde, A. Dodonova, D. Eliseev, M. Erdmann, P. Fackeldey, B. Fischer, T. Hebbeker, K. Hoepfner, F. Ivone, L. Mastrolorenzo, M. Merschmeyer, A. Meyer, G. Mocellin, S. Mondal, S. Mukherjee, D. Noll, A. Novak, A. Pozdnyakov, Y. Rath, H. Reithler, A. Schmidt, S. C. Schuler, A. Sharma, L. Vigilante, S. Wiedenbeck, S. Zaleski, C. Dziwok, G. Flügge, W. Haj Ahmad, O. Hlushchenko, T. Kress, A. Nowack, O. Pooth, D. Roy, A. Stahl, T. Ziemons, A. Zotz, H. Aarup Petersen, M. Aldaya Martin, P. Asmuss, S. Baxter, M. Bayatmakou, O. Behnke, A. Bermúdez Martínez, S. Bhattacharya, A. A. Bin Anuar, F. Blekman, K. Borras, D. Brunner, A. Campbell, A. Cardini, C. Cheng, F. Colombina, S. Consuegra Rodríguez, G. Correia Silva, V. Danilov, M. De Silva, L. Didukh, G. Eckerlin, D. Eckstein, L. I. Estevez Banos, O. Filatov, E. Gallo, A. Geiser, A. Giraldi, A. Grohsjean, M. Guthoff, A. Jafari, N. Z. Jomhari, A. Kasem, M. Kasemann, H. Kaveh, C. Kleinwort, R. Kogler, D. Krücker, W. Lange, K. Lipka, W. Lohmann, R. Mankel, I.-A. Melzer-Pellmann, M. Mendizabal Morentin, J. Metwally, A. B. Meyer, M. Meyer, J. Mnich, A. Mussgiller, A. Nürnberg, Y. Otarid, D. Pérez Adán, D. Pitzl, A. Raspereza, B. Ribeiro Lopes, J. Rübenach, A. Saggio, A. Saibel, M. Savitskyi, M. Scham, V. Scheurer, S. Schnake, P. Schütze, C. Schwanenberger, M. Shchedrolosiev, R. E. Sosa Ricardo, D. Stafford, N. Tonon, M. Van De Klundert, F. Vazzoler, R. Walsh, D. Walter, Q. Wang, Y. Wen, K. Wichmann, L. Wiens, C. Wissing, S. Wuchterl, R. Aggleton, S. Albrecht, S. Bein, L. Benato, P. Connor, K. De Leo, M. Eich, K. El Morabit, F. Feindt, A. Fröhlich, C. Garbers, E. Garutti, P. Gunnellini, M. Hajheidari, J. Haller, A. Hinzmann, G. Kasieczka, R. Klanner, T. Kramer, V. Kutzner, J. Lange, T. Lange, A. Lobanov, A. Malara, A. Mehta, A. Nigamova, K. J. Pena Rodriguez, M. Rieger, O. Rieger, P. Schleper, M. Schröder, J. Schwandt, J. Sonneveld, H. Stadie, G. Steinbrück, A. Tews, I. Zoi, J. Bechtel, S. Brommer, M. Burkart, E. Butz, R. Caspart, T. Chwalek, W. De Boer, A. Dierlamm, A. Droll, N. Faltermann, M. Giffels, J. O. Gosewisch, A. Gottmann, F. Hartmann, C. Heidecker, U. Husemann, P. Keicher, R. Koppenhöfer, S. Maier, M. Metzler, S. Mitra, Th. Müller, M. Neukum, G. Quast, K. Rabbertz, J. Rauser, D. Savoiu, M. Schnepf, D. Seith, I. Shvetsov, H. J. Simonis, R. Ulrich, J. Van Der Linden, R. F. Von Cube, M. Wassmer, M. Weber, S. Wieland, R. Wolf, S. Wozniewski, S. Wunsch, G. Anagnostou, G. Daskalakis, A. Kyriakis, A. Stakia, M. Diamantopoulou, D. Karasavvas, P. Kontaxakis, C. K. Koraka, A. Manousakis-Katsikakis, A. Panagiotou, I. Papavergou, N. Saoulidou, K. Theofilatos, E. Tziaferi, K. Vellidis, E. Vourliotis, G. Bakas, K. Kousouris, I. Papakrivopoulos, G. Tsipolitis, A. Zacharopoulou, K. Adamidis, I. Bestintzanos, I. Evangelou, C. Foudas, P. Gianneios, P. Katsoulis, P. Kokkas, N. Manthos, I. Papadopoulos, J. Strologas, M. Csanád, K. Farkas, M. M. A. Gadallah, S. Lökös, P. Major, K. Mandal, G. Pásztor, A. J. Rádl, O. Surányi, G. I. Veres, M. Bartók, G. Bencze, C. Hajdu, D. Horvath, F. Sikler, V. Veszpremi, S. Czellar, D. Fasanella, F. Fienga, J. Karancsi, J. Molnar, Z. Szillasi, D. Teyssier, P. Raics, Z. L. Trocsanyi, B. Ujvari, T. Csorgo, F. Nemes, T. Novak, S. Bansal, S. B. Beri, V. Bhatnagar, G. Chaudhary, S. Chauhan, N. Dhingra, R. Gupta, A. Kaur, H. Kaur, M. Kaur, P. Kumari, M. Meena, K. Sandeep, J. B. Singh, A. K. Virdi, A. Ahmed, A. Bhardwaj, B. C. Choudhary, M. Gola, S. Keshri, A. Kumar, M. Naimuddin, P. Priyanka, K. Ranjan, A. Shah, M. Bharti, R. Bhattacharya, S. Bhattacharya, D. Bhowmik, S. Dutta, S. Dutta, B. Gomber, M. Maity, P. Palit, P. K. Rout, G. Saha, B. Sahu, S. Sarkar, M. Sharan, P. K. Behera, S. C. Behera, P. Kalbhor, J. R. Komaragiri, D. Kumar, A. Muhammad, L. Panwar, R. Pradhan, P. R. Pujahari, A. Sharma, A. K. Sikdar, P. C. Tiwari, K. Naskar, T. Aziz, S. Dugad, M. Kumar, G. B. Mohanty, S. Banerjee, R. Chudasama, M. Guchait, S. Karmakar, S. Kumar, G. Majumder, K. Mazumdar, S. Mukherjee, S. Bahinipati, C. Kar, P. Mal, T. Mishra, V. K. Muraleedharan Nair Bindhu, A. Nayak, P. Saha, N. Sur, S. K. Swain, D. Vats, A. Alpana, S. Dube, B. Kansal, A. Laha, S. Pandey, A. Rastogi, S. Sharma, H. Bakhshiansohi, E. Khazaie, M. Zeinali, S. Chenarani, S. M. Etesami, M. Khakzad, M. Mohammadi Najafabadi, M. Grunewald, M. Abbrescia, R. Aly, C. Aruta, A. Colaleo, D. Creanza, N. De Filippis, M. De Palma, A. Di Florio, A. Di Pilato, W. Elmetenawee, F. Errico, L. Fiore, A. Gelmi, G. Iaselli, M. Ince, S. Lezki, G. Maggi, M. Maggi, I. Margjeka, V. Mastrapasqua, S. My, S. Nuzzo, A. Pellecchia, A. Pompili, G. Pugliese, D. Ramos, A. Ranieri, G. Selvaggi, L. Silvestris, F. M. Simone, Ü. Sözbilir, R. Venditti, P. Verwilligen, G. Abbiendi, C. Battilana, D. Bonacorsi, L. Borgonovi, L. Brigliadori, R. Campanini, P. Capiluppi, A. Castro, F. R. Cavallo, C. Ciocca, M. Cuffiani, G. M. Dallavalle, T. Diotalevi, F. Fabbri, A. Fanfani, P. Giacomelli, L. Giommi, C. Grandi, L. Guiducci, S. Lo Meo, L. Lunerti, S. Marcellini, G. Masetti, F. L. Navarria, A. Perrotta, F. Primavera, A. M. Rossi, T. Rovelli, G. P. Siroli, S. Albergo, S. Costa, A. Di Mattia, R. Potenza, A. Tricomi, C. Tuve, G. Barbagli, A. Cassese, R. Ceccarelli, V. Ciulli, C. Civinini, R. D’Alessandro, E. Focardi, G. Latino, P. Lenzi, M. Lizzo, M. Meschini, S. Paoletti, R. Seidita, G. Sguazzoni, L. Viliani, L. Benussi, S. Bianco, D. Piccolo, M. Bozzo, F. Ferro, R. Mulargia, E. Robutti, S. Tosi, A. Benaglia, G. Boldrini, F. Brivio, F. Cetorelli, F. De Guio, M. E. Dinardo, P. Dini, S. Gennai, A. Ghezzi, P. Govoni, L. Guzzi, M. T. Lucchini, M. Malberti, S. Malvezzi, A. Massironi, D. Menasce, L. Moroni, M. Paganoni, D. Pedrini, B. S. Pinolini, S. Ragazzi, N. Redaelli, T. Tabarelli de Fatis, D. Valsecchi, D. Zuolo, S. Buontempo, F. Carnevali, N. Cavallo, A. De Iorio, F. Fabozzi, A. O. M. Iorio, L. Lista, S. Meola, P. Paolucci, B. Rossi, C. Sciacca, P. Azzi, N. Bacchetta, D. Bisello, P. Bortignon, A. Bragagnolo, R. Carlin, P. Checchia, T. Dorigo, U. Dosselli, F. Gasparini, U. Gasparini, G. Grosso, L. Layer, E. Lusiani, M. Margoni, A. T. Meneguzzo, J. Pazzini, P. Ronchese, R. Rossin, F. Simonetto, G. Strong, M. Tosi, H. Yarar, M. Zanetti, P. Zotto, A. Zucchetta, G. Zumerle, C. Aimè, A. Braghieri, S. Calzaferri, D. Fiorina, P. Montagna, S. P. Ratti, V. Re, C. Riccardi, P. Salvini, I. Vai, P. Vitulo, P. Asenov, G. M. Bilei, D. Ciangottini, L. Fanò, M. Magherini, G. Mantovani, V. Mariani, M. Menichelli, F. Moscatelli, A. Piccinelli, M. Presilla, A. Rossi, A. Santocchia, D. Spiga, T. Tedeschi, P. Azzurri, G. Bagliesi, V. Bertacchi, L. Bianchini, T. Boccali, E. Bossini, R. Castaldi, M. A. Ciocci, V. D’Amante, R. Dell’Orso, M. R. Di Domenico, S. Donato, A. Giassi, F. Ligabue, E. Manca, G. Mandorli, D. Matos Figueiredo, A. Messineo, M. Musich, F. Palla, S. Parolia, G. Ramirez-Sanchez, A. Rizzi, G. Rolandi, S. Roy Chowdhury, A. Scribano, N. Shafiei, P. Spagnolo, R. Tenchini, G. Tonelli, N. Turini, A. Venturi, P. G. Verdini, P. Barria, M. Campana, F. Cavallari, D. Del Re, E. Di Marco, M. Diemoz, E. Longo, P. Meridiani, G. Organtini, F. Pandolfi, R. Paramatti, C. Quaranta, S. Rahatlou, C. Rovelli, F. Santanastasio, L. Soffi, R. Tramontano, N. Amapane, R. Arcidiacono, S. Argiro, M. Arneodo, N. Bartosik, R. Bellan, A. Bellora, J. Berenguer Antequera, C. Biino, N. Cartiglia, M. Costa, R. Covarelli, N. Demaria, B. Kiani, F. Legger, C. Mariotti, S. Maselli, E. Migliore, E. Monteil, M. Monteno, M. M. Obertino, G. Ortona, L. Pacher, N. Pastrone, M. Pelliccioni, M. Ruspa, K. Shchelina, F. Siviero, V. Sola, A. Solano, D. Soldi, A. Staiano, M. Tornago, D. Trocino, A. Vagnerini, S. Belforte, V. Candelise, M. Casarsa, F. Cossutti, A. Da Rold, G. Della Ricca, G. Sorrentino, S. Dogra, C. Huh, B. Kim, D. H. Kim, G. N. Kim, J. Kim, J. Lee, S. W. Lee, C. S. Moon, Y. D. Oh, S. I. Pak, S. Sekmen, Y. C. Yang, H. Kim, D. H. Moon, B. Francois, T. J. Kim, J. Park, S. Cho, S. Choi, B. Hong, K. Lee, K. S. Lee, J. Lim, J. Park, S. K. Park, J. Yoo, J. Goh, A. Gurtu, H. S. Kim, Y. Kim, J. Almond, J. H. Bhyun, J. Choi, S. Jeon, J. Kim, J. S. Kim, S. Ko, H. Kwon, H. Lee, S. Lee, B. H. Oh, M. Oh, S. B. Oh, H. Seo, U. K. Yang, I. Yoon, W. Jang, D. Y. Kang, Y. Kang, S. Kim, B. Ko, J. S. H. Lee, Y. Lee, J. A. Merlin, I. C. Park, Y. Roh, M. S. Ryu, D. Song, I. J. Watson, S. Yang, S. Ha, H. D. Yoo, M. Choi, H. Lee, Y. Lee, I. Yu, T. Beyrouthy, Y. Maghrbi, K. Dreimanis, V. Veckalns, M. Ambrozas, A. Carvalho Antunes De Oliveira, A. Juodagalvis, A. Rinkevicius, G. Tamulaitis, N. Bin Norjoharuddeen, Z. Zolkapli, J. F. Benitez, A. Castaneda Hernandez, H. A. Encinas Acosta, L. G. Gallegos Maríñez, M. León Coello, J. A. Murillo Quijada, A. Sehrawat, L. Valencia Palomo, G. Ayala, H. Castilla-Valdez, E. De La Cruz-Burelo, I. Heredia-De La Cruz, R. Lopez-Fernandez, C. A. Mondragon Herrera, D. A. Perez Navarro, R. Reyes-Almanza, A. Sánchez Hernández, S. Carrillo Moreno, C. Oropeza Barrera, F. Vazquez Valencia, I. Pedraza, H. A. Salazar Ibarguen, C. Uribe Estrada, J. Mijuskovic, N. Raicevic, D. Krofcheck, P. H. Butler, A. Ahmad, M. I. Asghar, A. Awais, M. I. M. Awan, M. Gul, H. R. Hoorani, W. A. Khan, M. A. Shah, M. Shoaib, M. Waqas, V. Avati, L. Grzanka, M. Malawski, H. Bialkowska, M. Bluj, B. Boimska, M. Górski, M. Kazana, M. Szleper, P. Zalewski, K. Bunkowski, K. Doroba, A. Kalinowski, M. Konecki, J. Krolikowski, M. Araujo, P. Bargassa, D. Bastos, A. Boletti, P. Faccioli, M. Gallinaro, J. Hollar, N. Leonardo, T. Niknejad, M. Pisano, J. Seixas, O. Toldaiev, J. Varela, P. Adzic, M. Dordevic, P. Milenovic, J. Milosevic, M. Aguilar-Benitez, J. Alcaraz Maestre, A. Álvarez Fernández, I. Bachiller, M. Barrio Luna, Cristina F. Bedoya, C. A. Carrillo Montoya, M. Cepeda, M. Cerrada, N. Colino, B. De La Cruz, A. Delgado Peris, J. P. Fernández Ramos, J. Flix, M. C. Fouz, O. Gonzalez Lopez, S. Goy Lopez, J. M. Hernandez, M. I. Josa, J. León Holgado, D. Moran, Á. Navarro Tobar, C. Perez Dengra, A. Pérez-Calero Yzquierdo, J. Puerta Pelayo, I. Redondo, L. Romero, S. Sánchez Navas, L. Urda Gómez, C. Willmott, J. F. de Trocóniz, B. Alvarez Gonzalez, J. Cuevas, C. Erice, J. Fernandez Menendez, S. Folgueras, I. Gonzalez Caballero, J. R. González Fernández, E. Palencia Cortezon, C. Ramón Álvarez, V. Rodríguez Bouza, A. Soto Rodríguez, A. Trapote, N. Trevisani, C. Vico Villalba, J. A. Brochero Cifuentes, I. J. Cabrillo, A. Calderon, J. Duarte Campderros, M. Fernandez, C. Fernandez Madrazo, P. J. Fernández Manteca, A. García Alonso, G. Gomez, C. Martinez Rivero, P. Martinez Ruiz del Arbol, F. Matorras, P. Matorras Cuevas, J. Piedra Gomez, C. Prieels, A. Ruiz-Jimeno, L. Scodellaro, I. Vila, J. M. Vizan Garcia, M. K. Jayananda, B. Kailasapathy, D. U. J. Sonnadara, D. D. C. Wickramarathna, W. G. D. Dharmaratna, K. Liyanage, N. Perera, N. Wickramage, T. K. Aarrestad, D. Abbaneo, J. Alimena, E. Auffray, G. Auzinger, J. Baechler, P. Baillon, D. Barney, J. Bendavid, M. Bianco, A. Bocci, C. Caillol, T. Camporesi, M. Capeans Garrido, G. Cerminara, N. Chernyavskaya, S. S. Chhibra, S. Choudhury, M. Cipriani, L. Cristella, D. d’Enterria, A. Dabrowski, A. David, A. De Roeck, M. M. Defranchis, M. Deile, M. Dobson, M. Dünser, N. Dupont, A. Elliott-Peisert, F. Fallavollita, A. Florent, L. Forthomme, G. Franzoni, W. Funk, S. Ghosh, S. Giani, D. Gigi, K. Gill, F. Glege, L. Gouskos, E. Govorkova, M. Haranko, J. Hegeman, V. Innocente, T. James, P. Janot, J. Kaspar, J. Kieseler, M. Komm, N. Kratochwil, C. Lange, S. Laurila, P. Lecoq, A. Lintuluoto, K. Long, C. Lourenço, B. Maier, L. Malgeri, S. Mallios, M. Mannelli, A. C. Marini, F. Meijers, S. Mersi, E. Meschi, F. Moortgat, M. Mulders, S. Orfanelli, L. Orsini, F. Pantaleo, E. Perez, M. Peruzzi, A. Petrilli, G. Petrucciani, A. Pfeiffer, M. Pierini, D. Piparo, M. Pitt, H. Qu, T. Quast, D. Rabady, A. Racz, G. Reales Gutiérrez, M. Rovere, H. Sakulin, J. Salfeld-Nebgen, S. Scarfi, C. Schäfer, M. Selvaggi, A. Sharma, P. Silva, W. Snoeys, P. Sphicas, S. Summers, K. Tatar, V. R. Tavolaro, D. Treille, P. Tropea, A. Tsirou, J. Wanczyk, K. A. Wozniak, W. D. Zeuner, L. Caminada, A. Ebrahimi, W. Erdmann, R. Horisberger, Q. Ingram, H. C. Kaestli, D. Kotlinski, M. Missiroli, L. Noehte, T. Rohe, K. Androsov, M. Backhaus, P. Berger, A. Calandri, A. De Cosa, G. Dissertori, M. Dittmar, M. Donegà, C. Dorfer, F. Eble, K. Gedia, F. Glessgen, T. A. Gómez Espinosa, C. Grab, D. Hits, W. Lustermann, A.-M. Lyon, R. A. Manzoni, L. Marchese, C. Martin Perez, M. T. Meinhard, F. Nessi-Tedaldi, J. Niedziela, F. Pauss, V. Perovic, S. Pigazzini, M. G. Ratti, M. Reichmann, C. Reissel, T. Reitenspiess, B. Ristic, D. Ruini, D. A. Sanz Becerra, V. Stampf, J. Steggemann, R. Wallny, C. Amsler, P. Bärtschi, C. Botta, D. Brzhechko, M. F. Canelli, K. Cormier, A. De Wit, R. Del Burgo, J. K. Heikkilä, M. Huwiler, W. Jin, A. Jofrehei, B. Kilminster, S. Leontsinis, S. P. Liechti, A. Macchiolo, P. Meiring, V. M. Mikuni, U. Molinatti, I. Neutelings, A. Reimers, P. Robmann, S. Sanchez Cruz, K. Schweiger, M. Senger, Y. Takahashi, C. Adloff, C. M. Kuo, W. Lin, A. Roy, T. Sarkar, S. S. Yu, L. Ceard, Y. Chao, K. F. Chen, P. H. Chen, P. s. Chen, H. Cheng, W.-S. Hou, Y.y. Li, R.-S. Lu, E. Paganis, A. Psallidas, A. Steen, H. y. Wu, E. Yazgan, P. r. Yu, B. Asavapibhop, C. Asawatangtrakuldee, N. Srimanobhas, F. Boran, S. Damarseckin, Z. S. Demiroglu, F. Dolek, I. Dumanoglu, E. Eskut, Y. Guler, E. Gurpinar Guler, C. Isik, O. Kara, A. Kayis Topaksu, U. Kiminsu, G. Onengut, K. Ozdemir, A. Polatoz, A. E. Simsek, B. Tali, U. G. Tok, S. Turkcapar, I. S. Zorbakir, G. Karapinar, K. Ocalan, M. Yalvac, B. Akgun, I. O. Atakisi, E. Gülmez, M. Kaya, O. Kaya, Ö. Özçelik, S. Tekten, E. A. Yetkin, A. Cakir, K. Cankocak, Y. Komurcu, S. Sen, S. Cerci, I. Hos, B. Isildak, B. Kaynak, S. Ozkorucuklu, H. Sert, C. Simsek, D. Sunar Cerci, C. Zorbilmez, B. Grynyov, L. Levchuk, D. Anthony, E. Bhal, S. Bologna, J. J. Brooke, A. Bundock, E. Clement, D. Cussans, H. Flacher, J. Goldstein, G. P. Heath, H. F. Heath, L. Kreczko, B. Krikler, S. Paramesvaran, S. Seif El Nasr-Storey, V. J. Smith, N. Stylianou, J. Taylor, K. Walkingshaw Pass, R. White, K. W. Bell, A. Belyaev, C. Brew, R. M. Brown, D. J. A. Cockerill, C. Cooke, K. V. Ellis, K. Harder, S. Harper, M.-L. Holmberg, J. Linacre, K. Manolopoulos, D. M. Newbold, E. Olaiya, D. Petyt, T. Reis, T. Schuh, C. H. Shepherd-Themistocleous, I. R. Tomalin, T. Williams, R. Bainbridge, P. Bloch, S. Bonomally, J. Borg, S. Breeze, O. Buchmuller, V. Cepaitis, G. S. Chahal, D. Colling, P. Dauncey, G. Davies, M. Della Negra, S. Fayer, G. Fedi, G. Hall, M. H. Hassanshahi, G. Iles, J. Langford, L. Lyons, A.-M. Magnan, S. Malik, A. Martelli, D. G. Monk, J. Nash, M. Pesaresi, B. C. Radburn-Smith, D. M. Raymond, A. Richards, A. Rose, E. Scott, C. Seez, A. Shtipliyski, A. Tapper, K. Uchida, T. Virdee, M. Vojinovic, N. Wardle, S. N. Webb, D. Winterbottom, K. Coldham, J. E. Cole, A. Khan, P. Kyberd, I. D. Reid, L. Teodorescu, S. Zahid, S. Abdullin, A. Brinkerhoff, B. Caraway, J. Dittmann, K. Hatakeyama, A. R. Kanuganti, B. McMaster, N. Pastika, M. Saunders, S. Sawant, C. Sutantawibul, J. Wilson, R. Bartek, A. Dominguez, R. Uniyal, A. M. Vargas Hernandez, A. Buccilli, S. I. Cooper, D. Di Croce, S. V. Gleyzer, C. Henderson, C. U. Perez, P. Rumerio, C. West, A. Akpinar, A. Albert, D. Arcaro, C. Cosby, Z. Demiragli, E. Fontanesi, D. Gastler, S. May, J. Rohlf, K. Salyer, D. Sperka, D. Spitzbart, I. Suarez, A. Tsatsos, S. Yuan, D. Zou, G. Benelli, B. Burkle, X. Coubez, D. Cutts, M. Hadley, U. Heintz, J. M. Hogan, T. Kwon, G. Landsberg, K. T. Lau, D. Li, M. Lukasik, J. Luo, M. Narain, N. Pervan, S. Sagir, F. Simpson, E. Usai, W. Y. Wong, X. Yan, D. Yu, W. Zhang, J. Bonilla, C. Brainerd, R. Breedon, M. Calderon De La Barca Sanchez, M. Chertok, J. Conway, P. T. Cox, R. Erbacher, G. Haza, F. Jensen, O. Kukral, R. Lander, M. Mulhearn, D. Pellett, B. Regnery, D. Taylor, Y. Yao, F. Zhang, M. Bachtis, R. Cousins, A. Datta, D. Hamilton, J. Hauser, M. Ignatenko, M. A. Iqbal, T. Lam, W. A. Nash, S. Regnard, D. Saltzberg, B. Stone, V. Valuev, Y. Chen, R. Clare, J. W. Gary, M. Gordon, G. Hanson, G. Karapostoli, O. R. Long, N. Manganelli, W. Si, S. Wimpenny, Y. Zhang, J. G. Branson, P. Chang, S. Cittolin, S. Cooperstein, N. Deelen, D. Diaz, J. Duarte, R. Gerosa, L. Giannini, J. Guiang, R. Kansal, V. Krutelyov, R. Lee, J. Letts, M. Masciovecchio, F. Mokhtar, M. Pieri, B. V. Sathia Narayanan, V. Sharma, M. Tadel, F. Würthwein, Y. Xiang, A. Yagil, N. Amin, C. Campagnari, M. Citron, G. Collura, A. Dorsett, V. Dutta, J. Incandela, M. Kilpatrick, J. Kim, B. Marsh, H. Mei, M. Oshiro, M. Quinnan, J. Richman, U. Sarica, F. Setti, J. Sheplock, P. Siddireddy, D. Stuart, S. Wang, A. Bornheim, O. Cerri, I. Dutta, J. M. Lawhorn, N. Lu, J. Mao, H. B. Newman, T. Q. Nguyen, M. Spiropulu, J. R. Vlimant, C. Wang, S. Xie, Z. Zhang, R. Y. Zhu, J. Alison, S. An, M. B. Andrews, P. Bryant, T. Ferguson, A. Harilal, C. Liu, T. Mudholkar, M. Paulini, A. Sanchez, W. Terrill, J. P. Cumalat, W. T. Ford, A. Hassani, G. Karathanasis, E. MacDonald, R. Patel, A. Perloff, C. Savard, N. Schonbeck, K. Stenson, K. A. Ulmer, S. R. Wagner, N. Zipper, J. Alexander, S. Bright-Thonney, X. Chen, Y. Cheng, D. J. Cranshaw, S. Hogan, J. Monroy, J. R. Patterson, D. Quach, J. Reichert, M. Reid, A. Ryd, W. Sun, J. Thom, P. Wittich, R. Zou, M. Albrow, M. Alyari, G. Apollinari, A. Apresyan, A. Apyan, L. A. T. Bauerdick, D. Berry, J. Berryhill, P. C. Bhat, K. Burkett, J. N. Butler, A. Canepa, G. B. Cerati, H. W. K. Cheung, F. Chlebana, K. F. Di Petrillo, J. Dickinson, V. D. Elvira, Y. Feng, J. Freeman, Z. Gecse, L. Gray, D. Green, S. Grünendahl, O. Gutsche, R. M. Harris, R. Heller, T. C. Herwig, J. Hirschauer, B. Jayatilaka, S. Jindariani, M. Johnson, U. Joshi, T. Klijnsma, B. Klima, K. H. M. Kwok, S. Lammel, D. Lincoln, R. Lipton, T. Liu, C. Madrid, K. Maeshima, C. Mantilla, D. Mason, P. McBride, P. Merkel, S. Mrenna, S. Nahn, J. Ngadiuba, V. Papadimitriou, K. Pedro, C. Pena, F. Ravera, A. Reinsvold Hall, L. Ristori, E. Sexton-Kennedy, N. Smith, A. Soha, L. Spiegel, J. Strait, L. Taylor, S. Tkaczyk, N. V. Tran, L. Uplegger, E. W. Vaandering, H. A. Weber, P. Avery, D. Bourilkov, L. Cadamuro, V. Cherepanov, R. D. Field, D. Guerrero, B. M. Joshi, M. Kim, E. Koenig, J. Konigsberg, A. Korytov, K. H. Lo, K. Matchev, N. Menendez, G. Mitselmakher, A. Muthirakalayil Madhu, N. Rawal, D. Rosenzweig, S. Rosenzweig, K. Shi, J. Wang, Z. Wu, E. Yigitbasi, X. Zuo, T. Adams, A. Askew, R. Habibullah, V. Hagopian, K. F. Johnson, R. Khurana, T. Kolberg, G. Martinez, H. Prosper, C. Schiber, O. Viazlo, R. Yohay, J. Zhang, M. M. Baarmand, S. Butalla, T. Elkafrawy, M. Hohlmann, R. Kumar Verma, D. Noonan, M. Rahmani, F. Yumiceva, M. R. Adams, H. Becerril Gonzalez, R. Cavanaugh, S. Dittmer, O. Evdokimov, C. E. Gerber, D. J. Hofman, A. H. Merrit, C. Mills, G. Oh, T. Roy, S. Rudrabhatla, M. B. Tonjes, N. Varelas, J. Viinikainen, X. Wang, Z. Ye, M. Alhusseini, K. Dilsiz, L. Emediato, R. P. Gandrajula, O. K. Köseyan, J.-P. Merlo, A. Mestvirishvili, J. Nachtman, H. Ogul, Y. Onel, A. Penzo, C. Snyder, E. Tiras, O. Amram, B. Blumenfeld, L. Corcodilos, J. Davis, A. V. Gritsan, S. Kyriacou, P. Maksimovic, J. Roskes, M. Swartz, T.Á. Vámi, A. Abreu, J. Anguiano, C. Baldenegro Barrera, P. Baringer, A. Bean, Z. Flowers, T. Isidori, S. Khalil, J. King, G. Krintiras, A. Kropivnitskaya, M. Lazarovits, C. Le Mahieu, C. Lindsey, J. Marquez, N. Minafra, M. Murray, M. Nickel, C. Rogan, C. Royon, R. Salvatico, S. Sanders, E. Schmitz, C. Smith, Q. Wang, Z. Warner, J. Williams, G. Wilson, S. Duric, A. Ivanov, K. Kaadze, D. Kim, Y. Maravin, T. Mitchell, A. Modak, K. Nam, F. Rebassoo, D. Wright, E. Adams, A. Baden, O. Baron, A. Belloni, S. C. Eno, N. J. Hadley, S. Jabeen, R. G. Kellogg, T. Koeth, Y. Lai, S. Lascio, A. C. Mignerey, S. Nabili, C. Palmer, M. Seidel, A. Skuja, L. Wang, K. Wong, D. Abercrombie, G. Andreassi, R. Bi, W. Busza, I. A. Cali, Y. Chen, M. D’Alfonso, J. Eysermans, C. Freer, G. Gomez-Ceballos, M. Goncharov, P. Harris, M. Hu, M. Klute, D. Kovalskyi, J. Krupa, Y.-J. Lee, C. Mironov, C. Paus, D. Rankin, C. Roland, G. Roland, Z. Shi, G. S. F. Stephans, J. Wang, Z. Wang, B. Wyslouch, R. M. Chatterjee, A. Evans, J. Hiltbrand, Sh. Jain, M. Krohn, Y. Kubota, J. Mans, M. Revering, R. Rusack, R. Saradhy, N. Schroeder, N. Strobbe, M. A. Wadud, K. Bloom, M. Bryson, S. Chauhan, D. R. Claes, C. Fangmeier, L. Finco, F. Golf, C. Joo, I. Kravchenko, I. Reed, J. E. Siado, G. R. Snow, W. Tabb, A. Wightman, F. Yan, A. G. Zecchinelli, G. Agarwal, H. Bandyopadhyay, L. Hay, I. Iashvili, A. Kharchilava, C. McLean, D. Nguyen, J. Pekkanen, S. Rappoccio, A. Williams, G. Alverson, E. Barberis, Y. Haddad, Y. Han, A. Hortiangtham, A. Krishna, J. Li, J. Lidrych, G. Madigan, B. Marzocchi, D. M. Morse, V. Nguyen, T. Orimoto, A. Parker, L. Skinnari, A. Tishelman-Charny, T. Wamorkar, B. Wang, A. Wisecarver, D. Wood, S. Bhattacharya, J. Bueghly, Z. Chen, A. Gilbert, T. Gunter, K. A. Hahn, Y. Liu, N. Odell, M. H. Schmitt, M. Velasco, R. Band, R. Bucci, M. Cremonesi, A. Das, N. Dev, R. Goldouzian, M. Hildreth, K. Hurtado Anampa, C. Jessop, K. Lannon, J. Lawrence, N. Loukas, L. Lutton, J. Mariano, N. Marinelli, I. Mcalister, T. McCauley, C. Mcgrady, K. Mohrman, C. Moore, Y. Musienko, R. Ruchti, A. Townsend, M. Wayne, M. Zarucki, L. Zygala, B. Bylsma, L. S. Durkin, B. Francis, C. Hill, M. Nunez Ornelas, K. Wei, B. L. Winer, B. R. Yates, F. M. Addesa, B. Bonham, P. Das, G. Dezoort, P. Elmer, A. Frankenthal, B. Greenberg, N. Haubrich, S. Higginbotham, A. Kalogeropoulos, G. Kopp, S. Kwan, D. Lange, D. Marlow, K. Mei, I. Ojalvo, J. Olsen, D. Stickland, C. Tully, S. Malik, S. Norberg, A. S. Bakshi, V. E. Barnes, R. Chawla, S. Das, L. Gutay, M. Jones, A. W. Jung, D. Kondratyev, A. M. Koshy, M. Liu, G. Negro, N. Neumeister, G. Paspalaki, S. Piperov, A. Purohit, J. F. Schulte, M. Stojanovic, J. Thieman, F. Wang, R. Xiao, W. Xie, J. Dolen, N. Parashar, D. Acosta, A. Baty, T. Carnahan, M. Decaro, S. Dildick, K. M. Ecklund, S. Freed, P. Gardner, F. J. M. Geurts, A. Kumar, W. Li, B. P. Padley, R. Redjimi, J. Rotter, W. Shi, A. G. Stahl Leiton, S. Yang, L. Zhang, Y. Zhang, A. Bodek, P. de Barbaro, R. Demina, J. L. Dulemba, C. Fallon, T. Ferbel, M. Galanti, A. Garcia-Bellido, O. Hindrichs, A. Khukhunaishvili, E. Ranken, R. Taus, G. P. Van Onsem, B. Chiarito, J. P. Chou, A. Gandrakota, Y. Gershtein, E. Halkiadakis, A. Hart, M. Heindl, O. Karacheban, I. Laflotte, A. Lath, R. Montalvo, K. Nash, M. Osherson, S. Salur, S. Schnetzer, S. Somalwar, R. Stone, S. A. Thayil, S. Thomas, H. Wang, H. Acharya, A. G. Delannoy, S. Fiorendi, S. Spanier, O. Bouhali, M. Dalchenko, A. Delgado, R. Eusebi, J. Gilmore, T. Huang, T. Kamon, H. Kim, S. Luo, S. Malhotra, R. Mueller, D. Overton, D. Rathjens, A. Safonov, N. Akchurin, J. Damgov, V. Hegde, S. Kunori, K. Lamichhane, S. W. Lee, T. Mengke, S. Muthumuni, T. Peltola, I. Volobouev, Z. Wang, A. Whitbeck, E. Appelt, S. Greene, A. Gurrola, W. Johns, A. Melo, K. Padeken, F. Romeo, P. Sheldon, S. Tuo, J. Velkovska, M. W. Arenton, B. Cardwell, B. Cox, G. Cummings, J. Hakala, R. Hirosky, M. Joyce, A. Ledovskoy, A. Li, C. Neu, C. E. Perez Lara, B. Tannenwald, S. White, N. Poudyal, S. Banerjee, K. Black, T. Bose, S. Dasu, I. De Bruyn, P. Everaerts, C. Galloni, H. He, M. Herndon, A. Herve, U. Hussain, A. Lanaro, A. Loeliger, R. Loveless, J. Madhusudanan Sreekala, A. Mallampalli, A. Mohammadi, D. Pinna, A. Savin, V. Shang, V. Sharma, W. H. Smith, D. Teague, S. Trembath-Reichert, W. Vetens, S. Afanasiev, V. Andreev, Yu. Andreev, T. Aushev, M. Azarkin, A. Babaev, A. Belyaev, V. Blinov, E. Boos, V. Borshch, D. Budkouski, V. Bunichev, O. Bychkova, V. Chekhovsky, R. Chistov, M. Danilov, A. Dermenev, T. Dimova, I. Dremin, M. Dubinin, L. Dudko, V. Epshteyn, G. Gavrilov, V. Gavrilov, S. Gninenko, V. Golovtcov, N. Golubev, I. Golutvin, I. Gorbunov, A. Gribushin, V. Ivanchenko, Y. Ivanov, V. Kachanov, L. Kardapoltsev, V. Karjavine, A. Karneyeu, V. Kim, M. Kirakosyan, D. Kirpichnikov, M. Kirsanov, V. Klyukhin, O. Kodolova, D. Konstantinov, V. Korenkov, A. Kozyrev, N. Krasnikov, E. Kuznetsova, A. Lanev, A. Litomin, N. Lychkovskaya, V. Makarenko, A. Malakhov, V. Matveev, V. Murzin, A. Nikitenko, S. Obraztsov, V. Okhotnikov, V. Oreshkin, A. Oskin, I. Ovtin, V. Palichik, P. Parygin, A. Pashenkov, V. Perelygin, M. Perfilov, S. Petrushanko, G. Pivovarov, S. Polikarpov, V. Popov, O. Radchenko, M. Savina, V. Savrin, V. Shalaev, S. Shmatov, S. Shulha, Y. Skovpen, S. Slabospitskii, I. Smirnov, V. Smirnov, D. Sosnov, A. Stepennov, V. Sulimov, E. Tcherniaev, A. Terkulov, O. Teryaev, M. Toms, A. Toropin, L. Uvarov, A. Uzunian, E. Vlasov, S. Volkov, A. Vorobyev, N. Voytishin, B. S. Yuldashev, A. Zarubin, I. Zhizhin, A. Zhokin, CMS Collaboration

**Affiliations:** 1 Yerevan Physics Institute, Yerevan, Armenia; 2 Institut für Hochenergiephysik, Vienna, Austria; 3 Universiteit Antwerpen, Antwerpen, Belgium; 4 Vrije Universiteit Brussel, Brussels, Belgium; 5 Université Libre de Bruxelles, Brussels, Belgium; 6 Ghent University, Ghent, Belgium; 7 Université Catholique de Louvain, Louvain-la-Neuve, Belgium; 8 Centro Brasileiro de Pesquisas Fisicas, Rio de Janeiro, Brazil; 9 Universidade do Estado do Rio de Janeiro, Rio de Janeiro, Brazil; 10 Universidade Estadual Paulista, Universidade Federal do ABC, São Paulo, Brazil; 11 Institute for Nuclear Research and Nuclear Energy, Bulgarian Academy of Sciences, Sofia, Bulgaria; 12 University of Sofia, Sofia, Bulgaria; 13 Beihang University, Beijing, China; 14 Department of Physics, Tsinghua University, Beijing, China; 15 Institute of High Energy Physics, Beijing, China; 16 State Key Laboratory of Nuclear Physics and Technology, Peking University, Beijing, China; 17 Sun Yat-Sen University, Guangzhou, China; 18 Institute of Modern Physics and Key Laboratory of Nuclear Physics and Ion-beam Application (MOE), Fudan University, Shanghai, China; 19 Zhejiang University, Hangzhou, Zhejiang China; 20 Universidad de Los Andes, Bogota, Colombia; 21 Universidad de Antioquia, Medellin, Colombia; 22 Faculty of Electrical Engineering, Mechanical Engineering and Naval Architecture, University of Split, Split, Croatia; 23 Faculty of Science, University of Split, Split, Croatia; 24 Institute Rudjer Boskovic, Zagreb, Croatia; 25 University of Cyprus, Nicosia, Cyprus; 26 Charles University, Prague, Czech Republic; 27 Escuela Politecnica Nacional, Quito, Ecuador; 28 Universidad San Francisco de Quito, Quito, Ecuador; 29 Academy of Scientific Research and Technology of the Arab Republic of Egypt, Egyptian Network of High Energy Physics, Cairo, Egypt; 30 Center for High Energy Physics (CHEP-FU), Fayoum University, El-Fayoum, Egypt; 31 National Institute of Chemical Physics and Biophysics, Tallinn, Estonia; 32 Department of Physics, University of Helsinki, Helsinki, Finland; 33 Helsinki Institute of Physics, Helsinki, Finland; 34 Lappeenranta-Lahti University of Technology, Lappeenranta, Finland; 35 IRFU, CEA, Université Paris-Saclay, Gif-sur-Yvette, France; 36 Laboratoire Leprince-Ringuet, CNRS/IN2P3, Ecole Polytechnique, Institut Polytechnique de Paris, Palaiseau, France; 37 Université de Strasbourg, CNRS, IPHC UMR 7178, Strasbourg, France; 38 Institut de Physique des 2 Infinis de Lyon (IP2I ), Villeurbanne, France; 39 Georgian Technical University, Tbilisi, Georgia; 40 RWTH Aachen University, I. Physikalisches Institut, Aachen, Germany; 41 RWTH Aachen University, III. Physikalisches Institut A, Aachen, Germany; 42 RWTH Aachen University, III. Physikalisches Institut B, Aachen, Germany; 43 Deutsches Elektronen-Synchrotron, Hamburg, Germany; 44 University of Hamburg, Hamburg, Germany; 45 Karlsruher Institut fuer Technologie, Karlsruhe, Germany; 46 Institute of Nuclear and Particle Physics (INPP), NCSR Demokritos, Aghia Paraskevi, Greece; 47 National and Kapodistrian University of Athens, Athens, Greece; 48 National Technical University of Athens, Athens, Greece; 49 University of Ioánnina, Ioánnina, Greece; 50 MTA-ELTE Lendület CMS Particle and Nuclear Physics Group, Eötvös Loránd University, Budapest, Hungary; 51 Wigner Research Centre for Physics, Budapest, Hungary; 52 Institute of Nuclear Research ATOMKI, Debrecen, Hungary; 53 Institute of Physics, University of Debrecen, Debrecen, Hungary; 54 Karoly Robert Campus, MATE Institute of Technology, Gyongyos, Hungary; 55 Panjab University, Chandigarh, India; 56 University of Delhi, Delhi, India; 57 Saha Institute of Nuclear Physics, HBNI, Kolkata, India; 58 Indian Institute of Technology Madras, Madras, India; 59 Bhabha Atomic Research Centre, Mumbai, India; 60 Tata Institute of Fundamental Research-A, Mumbai, India; 61 Tata Institute of Fundamental Research-B, Mumbai, India; 62 National Institute of Science Education and Research, An OCC of Homi Bhabha National Institute, Bhubaneswar, Odisha India; 63 Indian Institute of Science Education and Research (IISER), Pune, India; 64 Isfahan University of Technology, Isfahan, Iran; 65 Institute for Research in Fundamental Sciences (IPM), Tehran, Iran; 66 University College Dublin, Dublin, Ireland; 67 INFN Sezione di Bari, Università di Bari, Politecnico di Bari, Bari, Italy; 68 INFN Sezione di Bologna, Università di Bologna, Bologna, Italy; 69 INFN Sezione di Catania, Università di Catania, Catania, Italy; 70 INFN Sezione di Firenze, Università di Firenze, Florence, Italy; 71 INFN Laboratori Nazionali di Frascati, Frascati, Italy; 72 INFN Sezione di Genova, Università di Genova, Genoa, Italy; 73 INFN Sezione di Milano-Bicocca, Università di Milano-Bicocca, Milan, Italy; 74 INFN Sezione di Napoli, Università di Napoli ’Federico II’, Naples, Italy; Università della Basilicata, Potenza, Italy; Università G. Marconi, Rome, Italy; 75 INFN Sezione di Padova, Università di Padova, Padua, Italy; Università di Trento, Trento, Italy; 76 INFN Sezione di Pavia, Università di Pavia, Pavia, Italy; 77 INFN Sezione di Perugia, Università di Perugia, Perugia, Italy; 78 INFN Sezione di Pisa, Università di Pisa, Scuola Normale Superiore di Pisa, Pisa, Italy; Università di Siena, Siena, Italy; 79 INFN Sezione di Roma, Sapienza Università di Roma, Rome, Italy; 80 INFN Sezione di Torino, Università di Torino, Turin, Italy; Università del Piemonte Orientale, Novara, Italy; 81 INFN Sezione di Trieste, Università di Trieste, Trieste, Italy; 82 Kyungpook National University, Daegu, Korea; 83 Chonnam National University, Institute for Universe and Elementary Particles, Kwangju, Korea; 84 Hanyang University, Seoul, Korea; 85 Korea University, Seoul, Korea; 86 Department of Physics, Kyung Hee University, Seoul, Korea; 87 Sejong University, Seoul, Korea; 88 Seoul National University, Seoul, Korea; 89 University of Seoul, Seoul, Korea; 90 Department of Physics, Yonsei University, Seoul, Korea; 91 Sungkyunkwan University, Suwon, Korea; 92 College of Engineering and Technology, American University of the Middle East (AUM), Dasman, Kuwait; 93 Riga Technical University, Riga, Latvia; 94 Vilnius University, Vilnius, Lithuania; 95 National Centre for Particle Physics, Universiti Malaya, Kuala Lumpur, Malaysia; 96 Universidad de Sonora (UNISON), Hermosillo, Mexico; 97 Centro de Investigacion y de Estudios Avanzados del IPN, Mexico City, Mexico; 98 Universidad Iberoamericana, Mexico City, Mexico; 99 Benemerita Universidad Autonoma de Puebla, Puebla, Mexico; 100 University of Montenegro, Podgorica, Montenegro; 101 University of Auckland, Auckland, New Zealand; 102 University of Canterbury, Christchurch, New Zealand; 103 National Centre for Physics, Quaid-I-Azam University, Islamabad, Pakistan; 104 Faculty of Computer Science, Electronics and Telecommunications, AGH University of Science and Technology, Krakow, Poland; 105 National Centre for Nuclear Research, Swierk, Poland; 106 Institute of Experimental Physics, Faculty of Physics, University of Warsaw, Warsaw, Poland; 107 Laboratório de Instrumentação e Física Experimental de Partículas, Lisbon, Portugal; 108 VINCA Institute of Nuclear Sciences, University of Belgrade, Belgrade, Serbia; 109 Centro de Investigaciones Energéticas Medioambientales y Tecnológicas (CIEMAT), Madrid, Spain; 110 Universidad Autónoma de Madrid, Madrid, Spain; 111 Instituto Universitario de Ciencias y Tecnologías Espaciales de Asturias (ICTEA), Universidad de Oviedo, Oviedo, Spain; 112 Instituto de Física de Cantabria (IFCA), CSIC-Universidad de Cantabria, Santander, Spain; 113 University of Colombo, Colombo, Sri Lanka; 114 Department of Physics, University of Ruhuna, Matara, Sri Lanka; 115 CERN, European Organization for Nuclear Research, Geneva, Switzerland; 116 Paul Scherrer Institut, Villigen, Switzerland; 117 ETH Zurich-Institute for Particle Physics and Astrophysics (IPA), Zurich, Switzerland; 118 Universität Zürich, Zurich, Switzerland; 119 National Central University, Chung-Li, Taiwan; 120 National Taiwan University (NTU), Taipei, Taiwan; 121 Department of Physics, Faculty of Science, Chulalongkorn University, Bangkok, Thailand; 122 Physics Department, Science and Art Faculty, Çukurova University, Adana, Turkey; 123 Physics Department, Middle East Technical University, Ankara, Turkey; 124 Bogazici University, Istanbul, Turkey; 125 Istanbul Technical University, Istanbul, Turkey; 126 Istanbul University, Istanbul, Turkey; 127 Institute for Scintillation Materials of National Academy of Science of Ukraine, Kharkiv, Ukraine; 128 National Science Centre, Kharkiv Institute of Physics and Technology, Kharkiv, Ukraine; 129 University of Bristol, Bristol, UK; 130 Rutherford Appleton Laboratory, Didcot, UK; 131 Imperial College, London, UK; 132 Brunel University, Uxbridge, UK; 133 Baylor University, Waco, TX USA; 134 Catholic University of America, Washington, DC USA; 135 The University of Alabama, Tuscaloosa, AL USA; 136 Boston University, Boston, MA USA; 137 Brown University, Providence, RI USA; 138 University of California, Davis, Davis, CA USA; 139 University of California, Los Angeles, CA USA; 140 University of California, Riverside, Riverside, CA USA; 141 University of California, San Diego, La Jolla, CA USA; 142 Department of Physics, University of California, Santa Barbara, Santa Barbara, CA USA; 143 California Institute of Technology, Pasadena, CA USA; 144 Carnegie Mellon University, Pittsburgh, PA USA; 145 University of Colorado Boulder, Boulder, CO USA; 146 Cornell University, Ithaca, NY USA; 147 Fermi National Accelerator Laboratory, Batavia, IL USA; 148 University of Florida, Gainesville, FL USA; 149 Florida State University, Tallahassee, FL USA; 150 Florida Institute of Technology, Melbourne, FL USA; 151 University of Illinois at Chicago (UIC), Chicago, IL USA; 152 The University of Iowa, Iowa City, IA USA; 153 Johns Hopkins University, Baltimore, MD USA; 154 The University of Kansas, Lawrence, KS USA; 155 Kansas State University, Manhattan, KS USA; 156 Lawrence Livermore National Laboratory, Livermore, CA USA; 157 University of Maryland, College Park, MD USA; 158 Massachusetts Institute of Technology, Cambridge, MA USA; 159 University of Minnesota, Minneapolis, MN USA; 160 University of Nebraska-Lincoln, Lincoln, NE USA; 161 State University of New York at Buffalo, Buffalo, NY USA; 162 Northeastern University, Boston, MA USA; 163 Northwestern University, Evanston, IL USA; 164 University of Notre Dame, Notre Dame, IN USA; 165 The Ohio State University, Columbus, OH USA; 166 Princeton University, Princeton, NJ USA; 167 University of Puerto Rico, Mayaguez, PR USA; 168 Purdue University, West Lafayette, IN USA; 169 Purdue University Northwest, Hammond, IN USA; 170 Rice University, Houston, TX USA; 171 University of Rochester, Rochester, NY USA; 172 Rutgers, The State University of New Jersey, Piscataway, NJ USA; 173 University of Tennessee, Knoxville, TN USA; 174 Texas A &M University, College Station, TX USA; 175 Texas Tech University, Lubbock, TX USA; 176 Vanderbilt University, Nashville, TN USA; 177 University of Virginia, Charlottesville, VA USA; 178 Wayne State University, Detroit, MI USA; 179 University of Wisconsin-Madison, Madison, WI USA; 180 Institute or an international laboratory covered by a cooperation agreement with CERN, Geneva, Switzerland; 181 Yerevan State University, Yerevan, Armenia; 182 TU Wien, Vienna, Austria; 183 Institute of Basic and Applied Sciences, Faculty of Engineering, Arab Academy for Science, Technology and Maritime Transport, Alexandria, Egypt; 184 Université Libre de Bruxelles, Brussels, Belgium; 185 Universidade Estadual de Campinas, Campinas, Brazil; 186 Federal University of Rio Grande do Sul, Porto Alegre, Brazil; 187 The University of the State of Amazonas, Manaus, Brazil; 188 University of Chinese Academy of Sciences, Beijing, China; 189 UFMS, Nova Andradina, Brazil; 190 Nanjing Normal University Department of Physics, Nanjing, China; 191 The University of Iowa, Iowa City, IA USA; 192 University of Chinese Academy of Sciences, Beijing, China; 193 an institute or an international laboratory covered by a cooperation agreement with CERN, Geneva, Switzerland; 194 Helwan University, Cairo, Egypt; 195 Zewail City of Science and Technology, Zewail, Egypt; 196 British University in Egypt, Cairo, Egypt; 197 Purdue University, West Lafayette, IN USA; 198 Université de Haute Alsace, Mulhouse, France; 199 Ilia State University, Tbilisi, Georgia; 200 Erzincan Binali Yildirim University, Erzincan, Turkey; 201 RWTH Aachen University, III. Physikalisches Institut A, Aachen, Germany; 202 University of Hamburg, Hamburg, Germany; 203 Isfahan University of Technology, Isfahan, Iran; 204 Brandenburg University of Technology, Cottbus, Germany; 205 Forschungszentrum Jülich, Jülich, Germany; 206 CERN, European Organization for Nuclear Research, Geneva, Switzerland; 207 Physics Department, Faculty of Science, Assiut University, Assiut, Egypt; 208 Karoly Robert Campus, MATE Institute of Technology, Gyongyos, Hungary; 209 Institute of Physics, University of Debrecen, Debrecen, Hungary; 210 Institute of Nuclear Research ATOMKI, Debrecen, Hungary; 211 Universitatea Babes-Bolyai-Facultatea de Fizica, Cluj-Napoca, Romania; 212 MTA-ELTE Lendület CMS Particle and Nuclear Physics Group, Eötvös Loránd University, Budapest, Hungary; 213 Wigner Research Centre for Physics, Budapest, Hungary; 214 Punjab Agricultural University, Ludhiana, India; 215 UPES-University of Petroleum and Energy Studies, Dehradun, India; 216 Shoolini University, Solan, India; 217 University of Hyderabad, Hyderabad, India; 218 University of Visva-Bharati, Santiniketan, India; 219 Indian Institute of Science (IISc), Bangalore, India; 220 Indian Institute of Technology (IIT), Mumbai, India; 221 IIT Bhubaneswar, Bhubaneswar, India; 222 Institute of Physics, Bhubaneswar, India; 223 Deutsches Elektronen-Synchrotron, Hamburg, Germany; 224 Sharif University of Technology, Tehran, Iran; 225 Department of Physics, University of Science and Technology of Mazandaran, Behshahr, Iran; 226 Italian National Agency for New Technologies, Energy and Sustainable Economic Development, Bologna, Italy; 227 Centro Siciliano di Fisica Nucleare e di Struttura Della Materia, Catania, Italy; 228 Scuola Superiore Meridionale, Università di Napoli ’Federico II’, Naples, Italy; 229 Università di Napoli ’Federico II’, Naples, Italy; 230 Consiglio Nazionale delle Ricerche-Istituto Officina dei Materiali, Perugia, Italy; 231 Consejo Nacional de Ciencia y Tecnología, Mexico City, Mexico; 232 IRFU, CEA, Université Paris-Saclay, Gif-sur-Yvette, France; 233 Faculty of Physics, University of Belgrade, Belgrade, Serbia; 234 Trincomalee Campus, Eastern University, Nilaveli, Sri Lanka; 235 INFN Sezione di Pavia, Università di Pavia, Pavia, Italy; 236 National and Kapodistrian University of Athens, Athens, Greece; 237 Ecole Polytechnique Fédérale Lausanne, Lausanne, Switzerland; 238 Universität Zürich, Zurich, Switzerland; 239 Stefan Meyer Institute for Subatomic Physics, Vienna, Austria; 240 Laboratoire d’Annecy-le-Vieux de Physique des Particules, IN2P3-CNRS, Annecy-le-Vieux, France; 241 Şırnak University, Sirnak, Turkey; 242 Near East University, Research Center of Experimental Health Science, Mersin, Turkey; 243 Konya Technical University, Konya, Turkey; 244 Izmir Bakircay University, Izmir, Turkey; 245 Adiyaman University, Adiyaman, Turkey; 246 Necmettin Erbakan University, Konya, Turkey; 247 Bozok Universitetesi Rektörlügü, Yozgat, Turkey; 248 Marmara University, Istanbul, Turkey; 249 Milli Savunma University, Istanbul, Turkey; 250 Kafkas University, Kars, Turkey; 251 Istanbul Bilgi University, Istanbul, Turkey; 252 Hacettepe University, Ankara, Turkey; 253 Istanbul University-Cerrahpasa, Faculty of Engineering, Istanbul, Turkey; 254 Ozyegin University, Istanbul, Turkey; 255 Vrije Universiteit Brussel, Brussels, Belgium; 256 Rutherford Appleton Laboratory, Didcot, UK; 257 School of Physics and Astronomy, University of Southampton, Southampton, UK; 258 IPPP Durham University, Durham, UK; 259 Faculty of Science, Monash University, Clayton, Australia; 260 Università di Torino, Turin, Italy; 261 Bethel University, St. Paul, MN USA; 262 Karamanoğlu Mehmetbey University, Karaman, Turkey; 263 California Institute of Technology, Pasadena, CA USA; 264 United States Naval Academy, Annapolis, MD USA; 265 Ain Shams University, Cairo, Egypt; 266 Bingol University, Bingol, Turkey; 267 Georgian Technical University, Tbilisi, Georgia; 268 Sinop University, Sinop, Turkey; 269 Erciyes University, Kayseri, Turkey; 270 Institute of Modern Physics and Key Laboratory of Nuclear Physics and Ion-beam Application (MOE), Fudan University, Shanghai, China; 271 Texas A &M University at Qatar, Doha, Qatar; 272 Kyungpook National University, Daegu, Korea; 273 another institute or international laboratory covered by a cooperation agreement with CERN, Geneva, Switzerland; 274 Istanbul University, Istanbul, Turkey; 275 Yerevan Physics Institute, Yerevan, Armenia; 276 University of Florida, Gainesville, FL USA; 277 Imperial College, London, UK; 278 University of Rochester, Rochester, NY USA; 279 Baylor University, Waco, TX USA; 280 INFN Sezione di Torino, Università di Torino, Turin, Italy, Università del Piemonte Orientale, Novara, Italy; 281 Institute of Nuclear Physics of the Uzbekistan Academy of Sciences, Tashkent, Uzbekistan; 282 CERN, 1211 Geneva 23, Switzerland

## Abstract

A search is reported for pairs of light Higgs bosons (${\textrm{H}} _1$) produced in supersymmetric cascade decays in final states with small missing transverse momentum. A data set of LHC $\hbox {pp}$ collisions collected with the CMS detector at $\sqrt{s}=13\,\text {TeV}$ and corresponding to an integrated luminosity of 138$\,\text {fb}^{-1}$ is used. The search targets events where both ${\textrm{H}} _1$ bosons decay into  pairs that are reconstructed as large-radius jets using substructure techniques. No evidence is found for an excess of events beyond the background expectations of the standard model (SM). Results from the search are interpreted in the next-to-minimal supersymmetric extension of the SM, where a “singlino” of small mass leads to squark and gluino cascade decays that can predominantly end in a highly Lorentz-boosted singlet-like ${\textrm{H}} _1$ and a singlino-like neutralino of small transverse momentum. Upper limits are set on the product of the squark or gluino pair production cross section and the square of the  branching fraction of the ${\textrm{H}} _1$ in a benchmark model containing almost mass-degenerate gluinos and light-flavour squarks. Under the assumption of an SM-like  branching fraction, ${\textrm{H}} _1$ bosons with masses in the range 40–120$\,\text {GeV}$ arising from the decays of squarks or gluinos with a mass of 1200–2500$\,\text {GeV}$ are excluded at 95% confidence level.

## Introduction

**Fig. 1:**
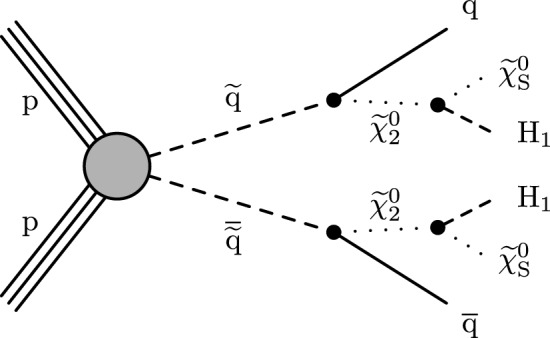
Diagram of squark pair production and subsequent cascade decay in the benchmark signal model. The particle $\tilde{\chi }^{0}_{2}$   is the next-to-LSP, $\tilde{\chi }_{\text {S}}^0$ is the singlino-like LSP, and ${\textrm{H}} _1$ is the *CP*-even singlet-like Higgs boson

This paper presents a search for pairs of light Higgs bosons (${\textrm{H}} _1$) produced in supersymmetric (SUSY) [1–8] cascade decays in final states with small missing transverse momentum ($p_{\textrm{T}} ^\text {miss}$). Such events can arise from the pair production of squarks (${\tilde{\textrm{q}}}$) and gluinos (${\tilde{\textrm{g}}}$) in the next-to-minimal supersymmetric extension of the standard model (SM) [9] when the lightest SUSY particle (LSP) is a singlino-like neutralino ($\tilde{\chi }_{\text {S}}^0$) of small mass [10]. The $\tilde{\chi }_{\text {S}}^0$ mass eigenstate is dominated by the singlino component and has only small couplings to other SUSY particles, suppressing direct squark or gluino decays to the $\tilde{\chi }_{\text {S}}^0$. Squarks and gluinos decay via the next-to-LSP $\tilde{\chi }^{0}_{2}$   into a $\tilde{\chi }_{\text {S}}^0$ and a Higgs, Z, or W  boson [10, 11]. The case of a singlet-like *CP*-even ${\textrm{H}} _1$, shown in Fig. 1, is the focus of this search. When the $\tilde{\chi }_{\text {S}}^0$ has a far smaller mass than the ${\textrm{H}} _1$ and the phase space for the decay $\tilde{\chi }^{0}_{2}\rightarrow {\textrm{H}} _1 +\tilde{\chi }_{\text {S}}^0$ is small, the ${\textrm{H}} _1$ carries much larger momentum than the $\tilde{\chi }_{\text {S}}^0$. In such $p_{\textrm{T}} ^\text {miss}$-suppressed scenarios, the key signature for the pair production of squarks and gluinos is a pair of Lorentz-boosted ${\textrm{H}} _1$ bosons.

This search targets events with two highly boosted ${\textrm{H}} _1$ bosons that decay into  pairs that are reconstructed as large-radius jets using substructure techniques. This is the first search at the LHC to focus on this type of event, where particles invisible to the detector have only small transverse momentum ($p_{\textrm{T}}$) and therefore the events are not selected by searches requiring significant $p_{\textrm{T}} ^\text {miss}$  [10, 12]. Previous searches by the ATLAS and CMS experiments with similar final states have considered events with two boosted SM Higgs bosons and large values of $p_{\textrm{T}} ^\text {miss}$  [13, 14], or two SM Higgs bosons in resolved final states where each of the four b quarks is reconstructed as a separate jet, with either small [15] or large [14–17] values of $p_{\textrm{T}} ^\text {miss}$. This search uses data from $\hbox {pp}$ collisions collected by the CMS detector at $\sqrt{s}=13\,\text {TeV}$ during 2016–2018, corresponding to an integrated luminosity of 138$\,\text {fb}^{-1}$  [18–20].

## Benchmark signal model

A benchmark signal model is established following the work of Ellwanger and Teixeira [10, 11]. The eight first- and second-generation squarks are assumed mass-degenerate at the mass $m_{\text {SUSY}}$, and the gluino mass is set at 1% larger. The small gluino-squark mass gap means that the kinematics of the final-state particles are very similar in the ${\tilde{\textrm{q}}}{\tilde{\textrm{q}}}$, ${\tilde{\textrm{q}}}{\tilde{\textrm{g}}}$, and ${\tilde{\textrm{g}}}{\tilde{\textrm{g}}}$ production modes, as little momentum is transferred to the quark in the ${\tilde{\textrm{g}}}\rightarrow {\tilde{\textrm{q}}}+{\textrm{q}}$ decay. All SUSY particles other than gluinos and those shown in Fig. 1 are assumed decoupled.

This search targets squarks and gluinos with $m_{\text {SUSY}} > 1200 \,\text {GeV}$. Less massive squarks and gluinos can be probed by $p_{\textrm{T}} ^\text {miss}$-based searches, owing to their larger pair-production cross sections [12]. Smaller $m_{\text {SUSY}}$ values can also lead to smaller $p_{\textrm{T}}$ of the ${\textrm{H}} _1$ than is necessary for the  pair to be merged in a single jet. The cross sections ($\sigma$) for the signal probed in this search, calculated at next-to-leading order (NLO) accuracy in the strong coupling constant ($\alpha _\textrm{S}$) including approximate next-to-NLO (NNLO) corrections and next-to-next-to-leading logarithmic (NNLL) soft gluon corrections [21–29], are shown in Table 1.

**Table 1:** Inclusive pair-production cross sections calculated at approximately NNLO and NNLL in $\alpha _\textrm{S}$  [21–29] for squark mass $m_{\text {SUSY}}$ and gluino mass 1% larger. The quoted uncertainty is obtained from variations in the choice of scales, parton distribution functions, and $\alpha _\textrm{S}$

$m_{\text {SUSY}}$ ($\text {GeV}$)	$\sigma (\hbox {pp} \rightarrow {\tilde{\textrm{q}}}{\tilde{\textrm{q}}},{\tilde{\textrm{q}}}{\tilde{\textrm{g}}},{\tilde{\textrm{g}}}{\tilde{\textrm{g}}})$ [fb]	Uncertainty (%)
1200	580	8
1600	69	9
2000	10	11
2200	4.1	13
2400	1.6	14
2600	0.67	16
2800	0.27	18

The values considered of the ${\textrm{H}} _1$ mass ($m_{{\textrm{H}} _1}$) and the corresponding  branching fractions ($\mathcal {B}$) are shown in Table 2. Only events where both ${\textrm{H}} _1$ bosons decay into  pairs are used as signal. The $\mathcal {B}$ values are chosen to be those of an SM-like Higgs boson (${\textrm{H}} _{\text {SM}}$) of the corresponding mass [10], as calculated using $\textsc {HDECAY}$ 6.61 [30, 31]. The $\mathcal {B}$ values decrease for larger ${\textrm{H}} _1$ masses as the virtual ${\textrm{W}} {\textrm{W}} ^{*}$ and ${\textrm{Z}} {\textrm{Z}} ^{*}$ decay channels, both of which have sizeable leptonic branching fractions, become more accessible. The region $m_{{\textrm{H}} _1} < m_{{\textrm{Z}}}$ is therefore where the $p_{\textrm{T}} ^\text {miss}$-suppressed all-jet signature is of greatest experimental importance. Nevertheless, to preserve generality, this search attempts to probe as much of the region $m_{{\textrm{H}} _1} < 125\,\text {GeV}$ as possible.

**Table 2:** The $m_{{\textrm{H}} _1}$ values in this search and corresponding  branching fractions

$m_{{\textrm{H}} _1}$ [$\text {GeV}$]	30	35	40	50	60	70	80	90	100	110	120	125
	0.86	0.86	0.86	0.86	0.85	0.84	0.83	0.81	0.79	0.75	0.65	0.58

In addition to $m_{{\textrm{H}} _1}$ and $m_{\text {SUSY}}$, there are two other unknown masses in the benchmark model: those of the $\tilde{\chi }_{\text {S}}^0$ and the $\tilde{\chi }^{0}_{2}$. The corresponding degrees of freedom are parameterised by $R_{m} \equiv m_{{\textrm{H}} _1}/ m_{\tilde{\chi }^{0}_{2}}$ and $\varDelta _{m} \equiv m_{\tilde{\chi }^{0}_{2}}- m_{{\textrm{H}} _1}- m_{\tilde{\chi }_{\text {S}}^0}$. The $p_{\textrm{T}} ^\text {miss}$-suppressed signature arises for values of $R_{m}$ close to unity, provided $\varDelta _{m} >0$ to permit the $\tilde{\chi }^{0}_{2}\rightarrow {\textrm{H}} _1 +\tilde{\chi }_{\text {S}}^0$ decay. In this case, the phase space for the $\tilde{\chi }^{0}_{2}$decay is small and the $\tilde{\chi }_{\text {S}}^0$ has much smaller mass than the ${\textrm{H}} _1$, so the $\tilde{\chi }_{\text {S}}^0$ always carries much less momentum than the ${\textrm{H}} _1$. The $p_{\textrm{T}} ^\text {miss}$-suppressed signature probed in this search is representative of a significant part of the model parameter space since the momenta of reconstructed objects do not exhibit a strong dependence on $R_{m}$ and $\varDelta _{m}$ in the region $R_{m} >0.9$. Models with smaller $R_{m}$ can be probed by $p_{\textrm{T}} ^\text {miss}$-based searches [10, 12]. For the benchmark model, the values $R_{m} = 0.99$ and $\varDelta _{m} = 0.1\,\text {GeV}$ are assumed.

Branching fractions of unity are assumed for the decays ${\tilde{\textrm{q}}}\rightarrow {\textrm{q}} +\tilde{\chi }^{0}_{2}$ and $\tilde{\chi }^{0}_{2}\rightarrow {\textrm{H}} _1 +\tilde{\chi }_{\text {S}}^0$. In the $R_{m}$ and $\varDelta _{m}$ region of the benchmark model, this is true except where $m_{\tilde{\chi }^{0}_{2}} > m_{{\textrm{Z}}} + m_{\tilde{\chi }_{\text {S}}^0}$. In that case, the $\tilde{\chi }^{0}_{2}\rightarrow {\textrm{Z}} +\tilde{\chi }_{\text {S}}^0$ decay is permitted if the $\tilde{\chi }^{0}_{2}$   has a higgsino component [11]. However, the $\tilde{\chi }^{0}_{2}$   is expected to be mainly bino-like for relevant values of its mass [10]. For configurations where the ${\textrm{H}} _1$ mass is close to that of the ${\textrm{H}} _{\text {SM}}$, the decay $\tilde{\chi }^{0}_{2}\rightarrow {\textrm{H}} _{\text {SM}} +\tilde{\chi }_{\text {S}}^0$ is also possible. The signatures for such ${\textrm{H}} _1$ and ${\textrm{H}} _{\text {SM}}$ bosons are indistinguishable in this search. The assumption that the branching fraction for $\tilde{\chi }^{0}_{2}\rightarrow {\textrm{H}} _1 +\tilde{\chi }_{\text {S}}^0$ decay is 100% can therefore be relaxed to the assumption that the branching fractions to ${\textrm{H}} _1$ and ${\textrm{H}} _{\text {SM}}$ sum to unity.

## The CMS detector

The central feature of the CMS apparatus is a superconducting solenoid of $6\,{\textrm{m}}$ internal diameter, providing a magnetic field of $3.8\,{\textrm{T}}$. A silicon pixel and strip tracker, a lead tungstate crystal electromagnetic calorimeter, and a brass and scintillator hadron calorimeter, each composed of a barrel and two endcap sections, reside within the solenoid volume. Forward calorimeters extend the pseudorapidity ($\eta$) coverage provided by the barrel and endcap detectors. Muons are measured in gas-ionisation detectors embedded in the steel flux-return yoke outside the solenoid. Events of interest are selected using a two-tiered trigger system. The first level, composed of custom hardware processors, uses information from the calorimeters and muon detectors to select events at a rate of around $100\,{\textrm{kHz}}$ within a fixed latency of about 4$\,\mu \text {s}$  [32]. The second level, known as the high-level trigger, consists of a farm of processors running a version of the full event reconstruction software optimised for fast processing, and reduces the event rate to around $1\,{\textrm{kHz}}$ before data storage [33]. A more detailed description of the CMS detector, together with a definition of the coordinate system and the kinematic variables, can be found in Ref. [34].

## Event simulation

The primary background in this search originates from multijet production. Simulated multijet events are used to validate the multijet background estimation based on data (described in Sect. 6), but are not used for any of the final predictions. The remaining significant background is from events with vector bosons that decay into quark–antiquark pairs. Simulated events are used to determine the contributions from ${\textrm{t}}\bar{\textrm{t}}$, Z+jets, and W+jets production. The expected yields from all other SM sources of background are found to be negligible.

The multijet, Z+jets, and W+jets processes are simulated at leading order (LO) in perturbative quantum chromodynamics (QCD) using MadGraph 5_amc@nlo  2.4.2 [35] with up to four additional partons at the matrix element (ME) level. Simulated signal events for each pair of $m_{{\textrm{H}} _1}$ and $m_{\text {SUSY}}$ values of the benchmark model are generated at LO at the ME level with up to one additional parton using MadGraph 5_amc@nlo  2.3.3. The MLM [36] prescription is used to match partons from the LO ME calculations to those from the parton showers. Simulated ${\textrm{t}}\bar{\textrm{t}}$ events are produced at NLO in QCD at the ME level with the powheg $\text {v}2.0$ [37–40] generator. The NNPDF2.3, NNPDF3.0, and NNPDF3.1 [41–44] parton distribution functions (PDFs) are used for the signal, 2016 background, and 2017–2018 background simulations, respectively. The parton shower and hadronisation are performed via pythia  8.2 [45]. The CUETP8M1 [46, 47] tune is used for the signal and 2016 background simulations, while the CP5 tune [48] is used for the 2017 and 2018 background simulations. The cross section used to normalise the ${\textrm{t}}\bar{\textrm{t}}$ simulation is calculated at NNLO+NNLL in QCD [49], and those for Z+jets and W+jets are calculated at NNLO in QCD [50–52]. Additional $\hbox {pp}$ interactions within the same or nearby bunch crossings (pileup) are simulated for all events according to the distribution of the number of interactions observed in each bunch crossing [53]. The interactions of particles with the CMS detector are simulated using Geant4  [54].

## Object reconstruction and event selection

The data are collected using triggers based on the scalar sum of jet $p_{\textrm{T}}$ ($H_{\textrm{T}}$), with a requirement of $H_{\textrm{T}}>900\,\text {GeV}$ (2016) and $H_{\textrm{T}}>1050\,\text {GeV}$ (2017 and 2018). Events are reconstructed offline using a particle-flow (PF) algorithm [55] that reconstructs and identifies each individual particle (PF candidate) in an event using an optimised combination of information from the components of the CMS detector.

Jets are reconstructed by clustering the PF candidates using the anti-$k_{\textrm{T}}$ clustering algorithm [56], as implemented in the FastJet package [57]. A distance parameter of 0.4 or 0.8 is used for standard- and large-radius jets, referred to as AK4 and AK8 jets, respectively. The jet momentum is defined as the vectorial sum of all particle momenta in the jet. To mitigate the effect of pileup, constituent charged PF candidates identified to be originating from vertices other than the primary $\hbox {pp}$ interaction vertex are not used in the clustering algorithm. The primary vertex is taken to be the vertex corresponding to the hardest scattering in the event, evaluated using tracking information alone, as described in Section 9.4 of Ref. [58]. For AK4 jets, an offset correction is applied to correct for remaining pileup contributions. For AK8 jets, the pileup-per-particle identification algorithm [59, 60] is used to rescale the momenta of constituent neutral particles according to the probability they originated from the primary vertex. This probability is based on a local shape variable that distinguishes between collinear and soft diffuse distributions of the surrounding charged particles that are compatible with the primary vertex. For all jets, jet energy corrections are derived from simulation to bring the measured average response of jets to that of particle-level jets. In situ measurements of the momentum balance in dijet, photon+jet, Z+jet, and multijet events are used to account for any residual differences in jet energy scale and resolution between data and simulation [61, 62]. Additional criteria are imposed to reject jets from spurious sources, such as electronics noise and detector malfunctions [63, 64].

The identification of AK8 jets originating from two collimated b quarks (double-b tagging) is integral to the reconstruction of the ${\textrm{H}} _1$. A discriminant is calculated for each jet using a double-b tagging algorithm that combines tracking and vertexing information in a multivariate approach with no strong dependence on jet mass or $p_{\textrm{T}}$  [65].

**Fig. 2:**
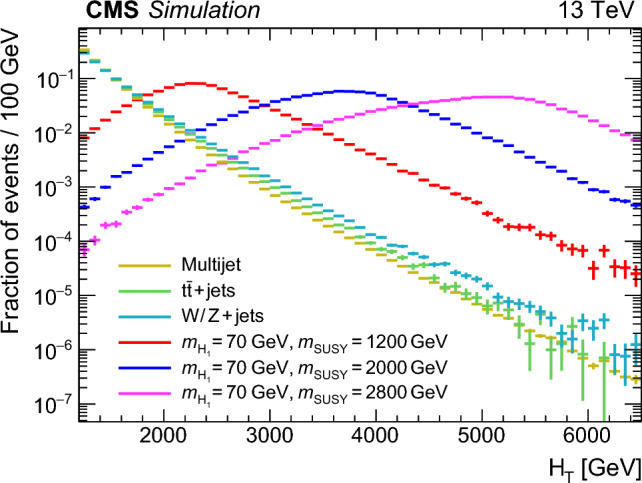
The $H_{\textrm{T}}$distribution in signal events with different values of $m_{\text {SUSY}}$, and in the simulated SM backgrounds, normalised to unit area. The uncertainties are statistical. All events satisfy the preselection

The event preselection requires two AK8 jets with $p_{\textrm{T}} > 170\,\text {GeV}$ and $|\eta | < 2.4$ (so that they are within the acceptance of the tracker). If there are more than two candidate AK8 jets, the two with the largest double-b  tag discriminants are selected as most likely to have originated from  decays. For the offline analysis, $H_{\textrm{T}}$is defined as the scalar $p_{\textrm{T}}$ sum of all AK4 jets with $p_{\textrm{T}} > 40\,\text {GeV}$ and $|\eta | < 3.0$, including AK4 jets with PF candidates clustered into AK8 jets. The $H_{\textrm{T}}$distributions for various simulated signal and background processes are shown in Fig. 2, after implementing all preselection requirements. Since the final state contains only jets, the average signal event $H_{\textrm{T}}$depends significantly on $m_{\text {SUSY}}$, and signal events with $m_{\text {SUSY}} > 1200 \,\text {GeV}$ tend to have $H_{\textrm{T}}>1500\,\text {GeV}$.

Additional requirements based on the expected kinematic properties of signal events are applied after the preselection. They define the kinematic event selection:

1. Both selected AK8 jets must have $p_{\textrm{T}} > 300\,\text {GeV}$ and $|\eta | < 2.4$, characteristic of the jets originating from  decay in signal events.
2. There must be at least one AK4 jet with $p_{\textrm{T}} > 300\,\text {GeV}$ and $|\eta | < 3.0$, characteristic of the quarks from squark decays in signal events. Such jets must be separated by $\varDelta R \equiv \sqrt{\smash [b]{(\varDelta \phi )^2 + (\varDelta \eta )^2}} > 1.4$ from both selected AK8 jets, to avoid being constructed from the same PF candidates.
3. The event $H_{\textrm{T}}$must exceed 1500$\,\text {GeV}$.

Although the offline $H_{\textrm{T}}$resolution is better than that of the trigger-level variable, the offline $H_{\textrm{T}}$threshold is comfortably above the trigger-level $H_{\textrm{T}}$requirements. The trigger efficiency for this analysis is measured using events collected with a single muon trigger with a muon $p_{\textrm{T}}$ threshold between 24 and 27$\,\text {GeV}$. The efficiency for each data-taking year is nearly 100%. For the 2018 data, the $|\eta |$ selection for the AK4 jets is reduced from 3.0 to 2.4 to avoid a region of the endcap electromagnetic calorimeters affected by large losses in crystal transparency, and therefore increased energy-equivalent electronics noise [66]. This change has a negligible effect on signal acceptance for all considered masses.

The fraction of signal events that satisfy the kinematic selection is essentially independent of $m_{{\textrm{H}} _1}$. It increases from about 60 to 80% as $m_{\text {SUSY}}$ increases from 1200 to 2000$\,\text {GeV}$, after which it remains approximately constant.

### Double-b tag based event selection

**Fig. 3:**
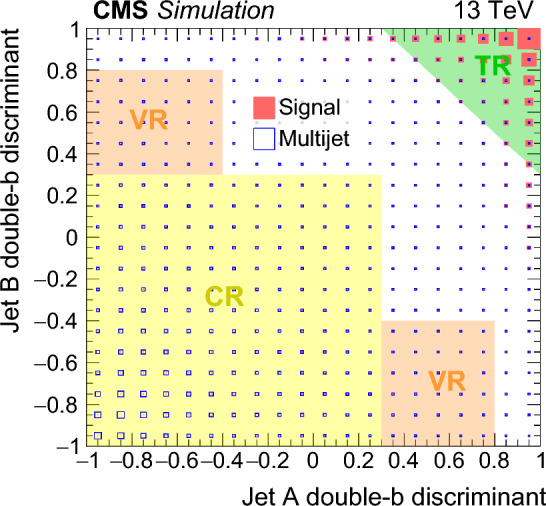
Distributions of simulated signal and multijet events in the 2D double-b  tag discriminant plane, where the fractions of events in each bin are represented by the areas of the filled red and open blue squares, respectively. The signal parameters are $m_{{\textrm{H}} _1} = 70\,\text {GeV}$ and $m_{\text {SUSY}} = 2000\,\text {GeV}$. The kinematic selection is implemented with the masses of the two AK8 jets required to be within the set of signal and sideband mass regions defined in Sect. 5.2. The green, yellow, and orange shaded areas represent the tag region (TR), control region (CR), and validation region (VR), respectively. Of the plotted signal events, 65% fall within the TR

The two AK8 jets that are classified as the  candidates in each event are randomly assigned the labels “A” and “B”. Their double-b  tag discriminants define a two-dimensional (2D) parameter space, shown with simulated signal and multijet event distributions in Fig. 3. The signal events are expected to contain two  decays and therefore accumulate in the region where both double-b  tag discriminants are large. The signal-enhanced tag region (TR) is defined as the region where the sum of the two double-b  tag discriminants exceeds 1.3, illustrated by the shaded triangle in Fig. 3. Two additional regions are defined in Fig. 3 for use in the multijet background estimation and validation: the control region (CR), a multijet-dominated region with negligible signal; and the validation region (VR), a more signal-like region where one of the two jets has a large double-b  tag discriminant. The VR is defined sufficiently far from the TR for the signal contamination to be negligible.

About 50% of the signal events that satisfy the kinematic selection populate the TR, with variation at the level of $\pm 10\%$ across the $m_{{\textrm{H}} _1}$ and $m_{\text {SUSY}}$ parameter space considered. Since the multijet background is dominated by light-flavour quark and gluon initiated jets, only about 3% of these events populate the TR. For the ${\textrm{t}}\bar{\textrm{t}}$, Z+jets, and W+jets backgrounds, the corresponding figures are 13, 6, and 3%, respectively.

### Soft-drop mass based signal and sideband regions

In signal events, both selected AK8 jets are likely to originate from  decays and therefore have a jet mass close to $m_{{\textrm{H}} _1}$. The multijet background has no resonant mass peak, while the other backgrounds are only expected to exhibit peaks near the known top quark and vector bosons masses, which means that an accurate reconstruction of the jet mass is important in distinguishing signal from background. The AK8 jet masses are evaluated using the “soft-drop” algorithm [67] (with a soft-drop threshold of $z_{\text {cut}} = 0.1$ and angular exponent of $\beta = 0$), in which wide-angle soft radiation is removed recursively from a jet. In signal events this algorithm achieves a relative jet mass resolution from 10% for $m_{{\textrm{H}} _1} =125\,\text {GeV}$ to 20% for $m_{{\textrm{H}} _1} =30\,\text {GeV}$.

**Fig. 4:**
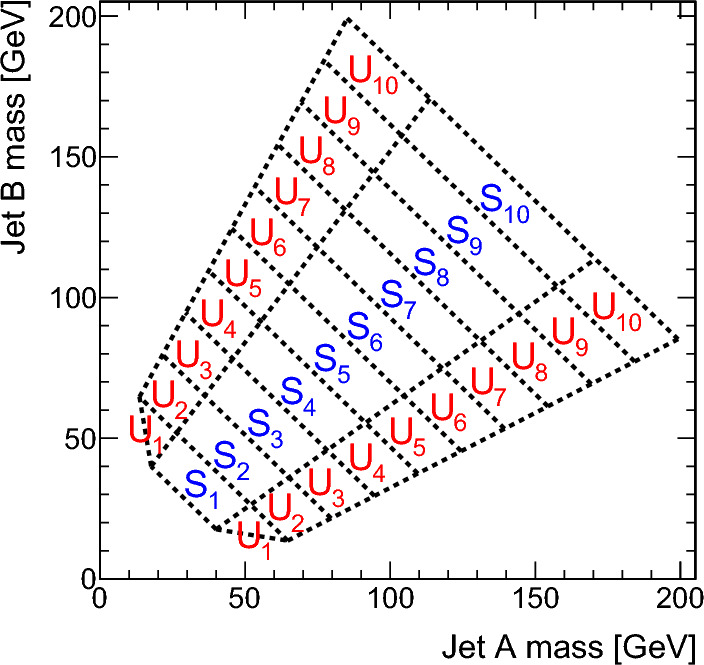
Map of mass regions used in the 2D soft-drop mass plane. The regions labelled $S_i$ are the signal mass regions, and the disjoint regions $U_{i}$ form the corresponding sidebands

The soft-drop masses of the two AK8 jets define a 2D parameter space, shown in Fig. 4, in which 10 signal regions ($S_i$) and 10 sideband regions ($U_{i}$) are defined. The $S_i$ contain events in which the two ${\textrm{H}} _1$-candidate jets have approximately the same soft-drop mass. The width of each $S_i$ corresponds to about four times the experimental soft-drop mass resolution for the relevant simulated value of $m_{{\textrm{H}} _1}$.

**Fig. 5:**
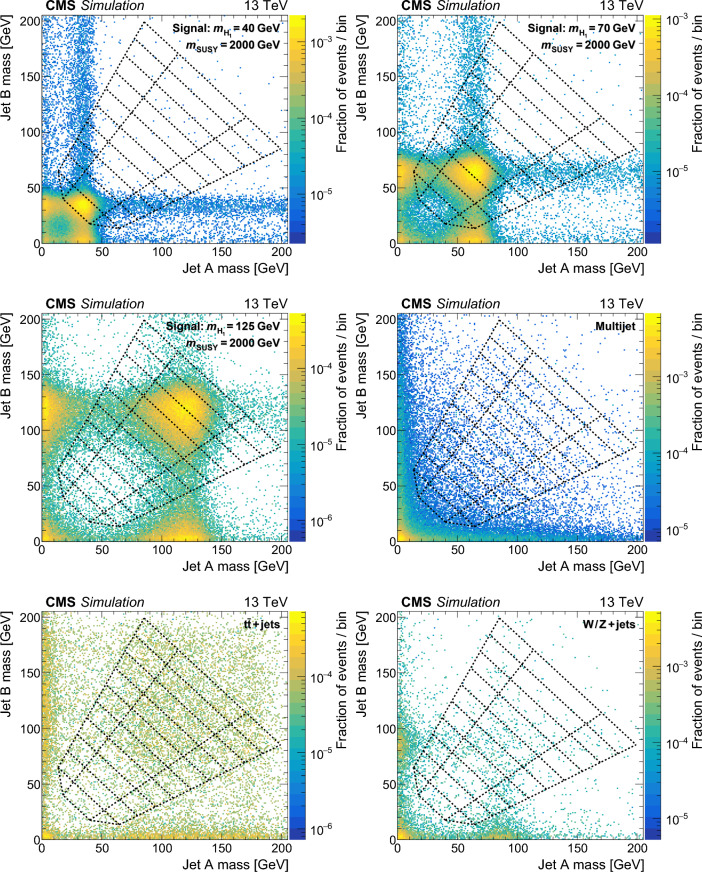
The normalised distribution of events in the 2D soft-drop mass plane overlaid by the map of mass regions. The upper left, upper right, and middle left panels correspond to signal events for $m_{\text {SUSY}} = 2000\,\text {GeV}$ and $m_{{\textrm{H}} _1}$ values of 40, 70, and 125$\,\text {GeV}$, respectively. The panels at middle right, lower left, and lower right correspond to simulated multijet, ${\textrm{t}}\bar{\textrm{t}}$, and vector boson backgrounds, respectively. All events satisfy the TR requirement and the kinematic selection

The event distributions for a set of signal models with different $m_{{\textrm{H}} _1}$ values are shown in Fig. 5, with the signal and sideband mass regions overlaid. The peaks in the signal distributions where one or both AK8 jets have a soft-drop mass close to zero result from a selected jet originating from a single parton or one of the  decays lying outside the acceptance of the jet reconstruction algorithm. The latter can happen when the angular separation of the b  quarks exceeds the AK8 jet distance parameter, or when the ratio of the b  quark $p_{\textrm{T}}$ values is larger than 9 (such that the softer b  quark would not satisfy the $z_{\text {cut}}$ threshold in the soft-drop algorithm). For signal models with $40< m_{{\textrm{H}} _1} < 125\,\text {GeV}$, $\approx \!50\%$ of the events that satisfy the kinematic and TR selection fall within any of the $S_i$. However, for $m_{{\textrm{H}} _1} < 35\,\text {GeV}$ the bulk of the distribution is lower in mass than $S_1$, leading to a rapid decrease in signal acceptance.

The distributions of background events are also shown in Fig. 5. The majority of multijet events contain at least one AK8 jet evaluated to have a small soft-drop mass, reflecting the characteristic one-prong structure of quark and gluon jets. After applying the kinematic and TR selection criteria, approximately 5% of multijet events fall within any of the $S_i$, with greater probability at small masses. For the vector boson and ${\textrm{t}}\bar{\textrm{t}}$ backgrounds the corresponding figures are 7 and 19%, respectively, concentrated in the $S_i$ corresponding to masses between the W  boson and top quark masses.

For each $S_i$ there are two corresponding sideband regions, $U_{i}$, used for the multijet background estimation described in Sect. 6. The sideband regions $U_{1}$ have a triangular form to avoid the region of very small soft-drop masses, where the density from multijet events increases sharply.

### Categorisation in $H_{\textrm{T}}$and expected yields

**Fig. 6:**
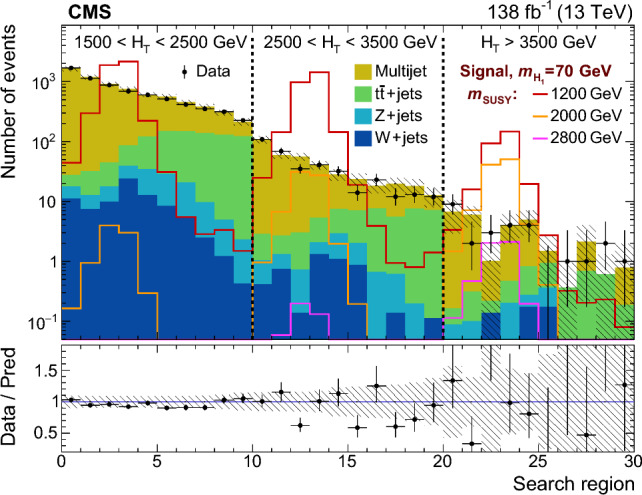
Observed and expected yields in the TR for each of the 30 search regions, summed over the three data-taking years. The multijet background is estimated from data using the method described in Sect. 6, while the other backgrounds are simulated. Example signal distributions are shown for $m_{{\textrm{H}} _1} = 70\,\text {GeV}$ and $m_{\text {SUSY}} =1200$, 2000, and 2800$\,\text {GeV}$. The error bars represent the statistical uncertainties and the hatched bands the systematic uncertainties

The selected events are classified according to three $H_{\textrm{T}}$ categories: 1500–2500, 2500–3500, and above $3500\,\text {GeV}$. Each $H_{\textrm{T}}$category is divided into the 10 mass signal regions $S_i$ defined in Fig. 4, resulting in a total of 30 search regions for each data-taking year. As can be seen in Fig. 6 for TR data summed over the three data-taking years, the search region yields can be visualised through a 30-bin histogram where bins 1–10 represent the $S_i$, in ascending order, for the first $H_{\textrm{T}}$category. The subsequent two sets of 10 bins represent the results for the second and third $H_{\textrm{T}}$categories. The primary background is from multijet events, estimated from data using the method described in Sect. 6. The expected contribution from ${\textrm{t}}\bar{\textrm{t}}$ events is also significant, particularly in the larger soft-drop mass regions populated by jets from hadronic top quark or W boson decays. The ${\textrm{t}}\bar{\textrm{t}}$ simulation is validated in a dedicated ${\textrm{t}}\bar{\textrm{t}}$-enriched control region in data. In Fig. 4 this is the triangular region of the parameter space with both jet masses below 200$\,\text {GeV}$ and above the upper boundary of mass region 10. The yields from Z+jets and W+jets production are small in comparison. All expected SM backgrounds tend to exhibit small values of $H_{\textrm{T}}$compared to signal.

The distributions in signal events for $m_{{\textrm{H}} _1} = 70\,\text {GeV}$ and $m_{\text {SUSY}} =1200$, 2000 and 2800$\,\text {GeV}$ are also shown in Fig. 6. Although the production cross section decreases quickly with increasing $m_{\text {SUSY}}$, the fraction of events in the larger-$H_{\textrm{T}}$categories increases. Within each $H_{\textrm{T}}$category, the distribution of events in the 10 $S_i$ bins is described by a peak with a width of about three bins, centred near the model value of $m_{{\textrm{H}} _1}$.

## Multijet background estimation from data

The mass sideband regions $U_{i}$ form a basis for using data to estimate the multijet background. The density of the multijet background is approximately uniform within each of the 10 mass regions (spanning $S_i$ and $U_{i}$ for each region *i* illustrated in Fig. 4). Apart from $U_{1}$, each $U_{i}$ is constructed to have the same area as $S_i$ such that the corresponding multijet yields, respectively denoted $\hat{U}_{i}$ and $\hat{S}_{i}$, are approximately equal. The observed ratios of $S_i$ to $U_{i}$ yields, $F_{i}$, are measured in CR data. The $F_{i}$ factors are found to be close to unity except for the $F_{1}$ values which are approximately 1.5.

The multijet background in the TR is estimated independently for each signal region $S_i$:

1 $$\begin{aligned} \hat{S}_{i}^{\text {TR}} = F_{i} \, \hat{U}_{i} ^{\text {TR}}, \end{aligned}$$

where $\hat{U}_{i} ^{\text {TR}}$ is the observed TR yield in sideband region $U_{i}$ after subtracting the contributions from the other simulated backgrounds. In rare cases where the prediction $\hat{S}_{i}^{\text {TR}}$ is negative, it is set equal to zero.

Since the $F_i$ factors are measured and applied in different regions of double-b  tag discriminant space, any correlation between the soft-drop mass and the double-b  tag discriminant of AK8 jets can bias the prediction of Eq. (1). Using a sample of data satisfying an alternative kinematic event selection with the requirement for one or more AK4 jets inverted, the variation of $F_i$ between the TR and the CR is found to be less than 10%.

**Fig. 7:**
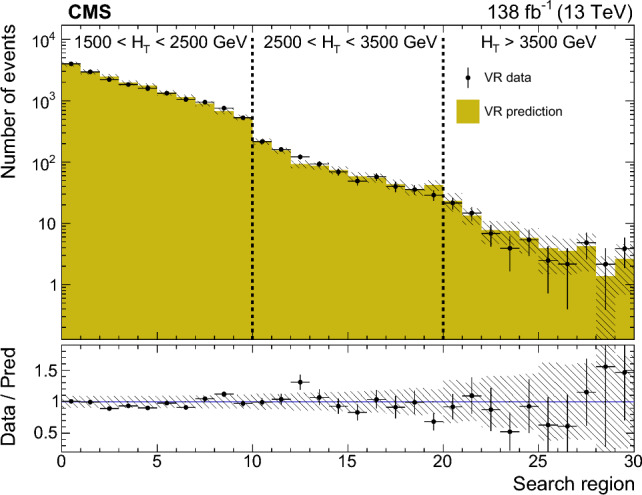
A comparison of the predicted and observed multijet yields in the validation region (VR), after subtraction of the other simulated backgrounds. The prediction is made separately for the three data-taking years, and the results are summed. The error bars on the data points represent their statistical uncertainties. The uncertainties in the predicted yields (statistical and systematic) are indicated by the hatched bands

The overall accuracy of the multijet estimation is assessed through closure tests. First the method is applied to simulated multijet events in the TR where, within statistical uncertainties, the predicted yields are consistent with the simulated yields for each data-taking year. Second the method is applied in the multijet-dominated VR data (defined in Fig. 3) by making the appropriate modification to Eq. (1): $\hat{S}_{i}^{\text {VR}} = F_i \, \hat{U}_{i} ^{\text {VR}}$. The resulting predicted and observed VR yields are consistent within uncertainties, as shown in Fig. 7. Based on the results of the closure tests, a systematic uncertainty of 15 (30%) is assigned in the lower two $H_{\textrm{T}}$categories (upper $H_{\textrm{T}}$category).

## Systematic uncertainties

The simulated events for signal and the ${\textrm{t}}\bar{\textrm{t}}$, Z+jets, and W+jets backgrounds are affected by various systematic uncertainties. The efficiency for tagging (mistagging) a jet originating from two b quarks (a light-flavour quark or gluon) is corrected to match that observed in data [65]. The uncertainty in this correction corresponds to $\approx \!10\%$ in the simulated signal and background yields. The uncertainties related to the jet energy corrections are applied to the jet properties in bins of $p_{\textrm{T}}$ and $\eta$. These uncertainties affect the event $H_{\textrm{T}}$, leading to an $\approx \!4\%$ migration of events between adjacent $H_{\textrm{T}}$categories. The uncertainty in the soft-drop mass scale in simulation relative to data leads to a migration of events between adjacent $S_i$ and $U_{i}$ regions of up to 10%. The uncertainty in the simulated soft-drop mass resolution affects the widths of the simulated mass peaks. This effect is larger for signal models with small $m_{{\textrm{H}} _1}$ and can reduce the $S_i$ selection efficiency by up to 20%.

The systematic uncertainties are assumed to be fully correlated among the data-taking years except for the 2016 double-b tagging uncertainties, which are assumed uncorrelated because the CMS pixel detector was upgraded prior to 2017 data-taking. Changing these correlation assumptions is found to have only a small effect on the final results. Systematic uncertainties related to integrated luminosity, pileup, PDFs, renormalisation and factorisation scales, modelling of initial-state radiation, and background cross sections were also evaluated, along with the statistical uncertainties in the simulation, and were found to make negligible contributions to the total uncertainty.

Systematic uncertainties in multijet yields arise from the systematic uncertainties in the $F_i$ factors. As described in Sect. 6, an uncertainty of $15\%$ is applied to the $F_i$ in the lower two $H_{\textrm{T}}$categories and $30\%$ in the upper $H_{\textrm{T}}$category, uncorrelated among different $F_i$. Except in the lowest $H_{\textrm{T}}$category, the total uncertainty in the multijet yield is dominated by the statistical uncertainty in $\hat{U}_{i} ^{\text {TR}}$.

## Results

**Fig. 8:**
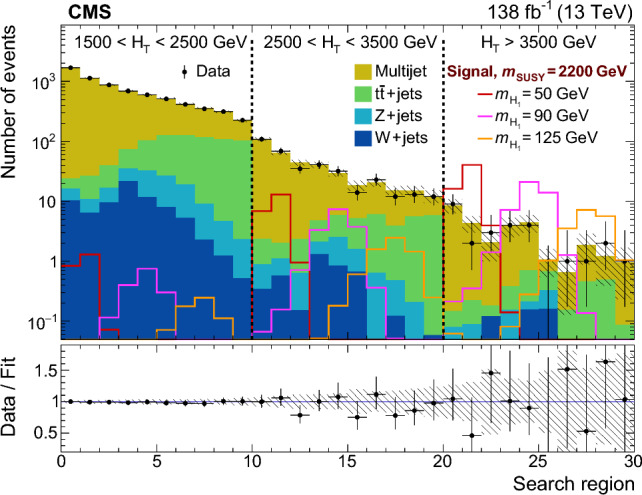
Yields in all search regions after the background-only fit, summed over the three data-taking years. Example signal contributions used in the signal+background fits are shown for $m_{\text {SUSY}} =2200\,\text {GeV}$, and $m_{{\textrm{H}} _1} =50$, 90, and 125$\,\text {GeV}$. The error bars represent the statistical uncertainties and the hatched bands the systematic uncertainties

Binned maximum likelihood fits to the data in all 30 search regions $S_i$ for each data-taking year are carried out under background-only and signal+background hypotheses. The corresponding sideband regions $U_{i}$ are fitted simultaneously, thereby constraining the multijet contributions to the search region yields through Eq. (1). The likelihood functions are defined through the product of $90 \times 2$ Poisson distributions [68], one for each search region and one for each sideband region, with additional constraint terms for the “nuisance” parameters that account for the systematic uncertainties summarised in Sect. 7. Figure 8 compares the result of the background-only fit to the yields in the search regions for the combination of 2016, 2017, and 2018 data. There is no evidence for deviations of the data from the fitted background. The values and uncertainties of most nuisance parameters are unchanged in the fit, but the ones corresponding to the $F_i$ are constrained through Eq. (1) when the yields $\hat{S}_{i}^{\text {TR}}$ and $\hat{U}_{i} ^{\text {TR}}$ are sufficiently large.

**Fig. 9:**
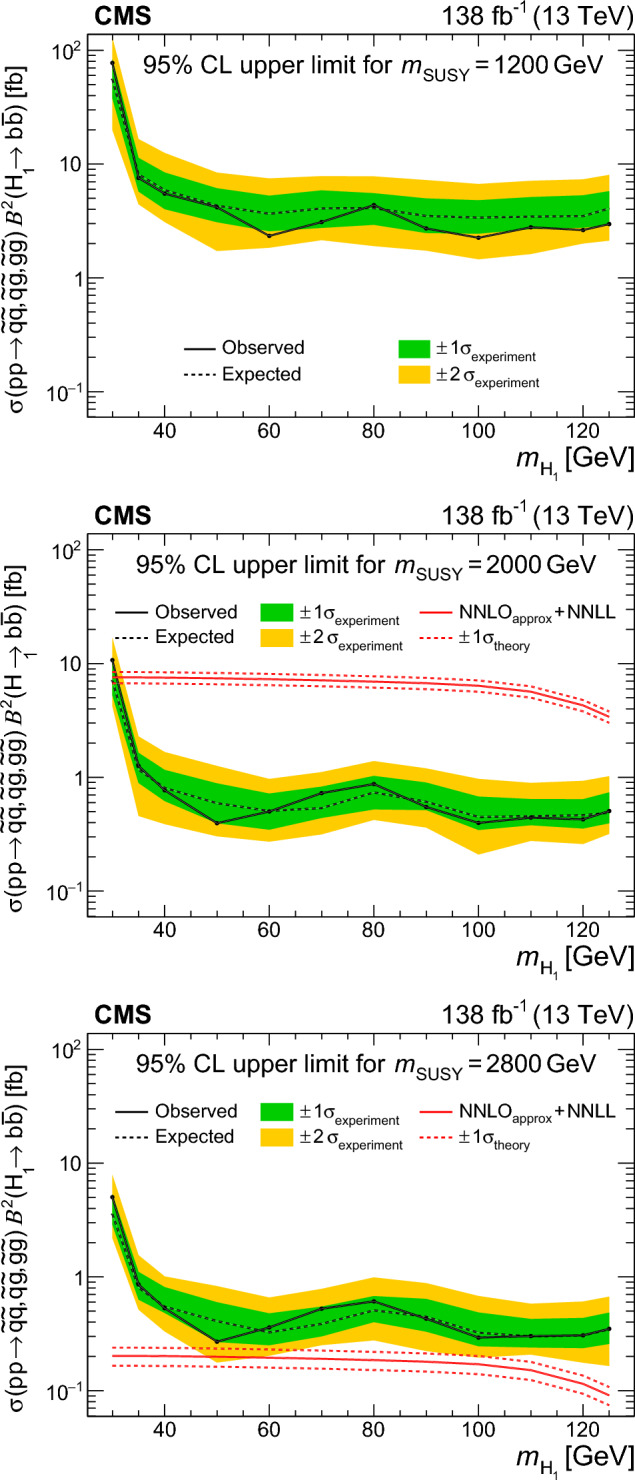
Upper limits at 95% $\textrm{CL}$on $\sigma \mathcal {B} ^2$ as a function of $m_{{\textrm{H}} _1}$, for $m_{\text {SUSY}}$ values of 1200 (upper), 2000 (middle), and 2800$\,\text {GeV}$ (lower). The solid and dashed black lines indicate the observed and median expected limits, respectively. The inner (green) band and the outer (yellow) band indicate the regions containing 68 and 95%, respectively, of the distribution of limits expected under the background-only hypothesis. The solid and dashed red lines show the theoretical value of $\sigma \mathcal {B} ^2$ and its uncertainty [21–30]. In the upper plot, these $\sigma \mathcal {B} ^2$ values are beyond the maximum of the vertical axis

Signal+background fits are used to set 95% confidence level ($\textrm{CL}$) upper limits on the product $\sigma \mathcal {B} ^2$ for the mass points in the benchmark signal model. The limits are set using the modified frequentist $\textrm{CL}_{{s}}$ criterion [69, 70], with the profile likelihood ratio as test statistic [68]. The observed and expected 95% $\textrm{CL}$upper limits on $\sigma \mathcal {B} ^2$ are shown in Fig. 9, as functions of $m_{{\textrm{H}} _1}$ for constant $m_{\text {SUSY}}$. The upper limits are weaker for models with $m_{{\textrm{H}} _1} <35\,\text {GeV}$, for which the signal-event distribution in the 2D soft-drop mass plane peaks outside the signal regions. The limits have no significant dependence on $m_{\text {SUSY}}$ for models with $m_{\text {SUSY}} >2000\,\text {GeV}$, whose signal events mostly populate the upper $H_{\textrm{T}}$category (as shown in Fig. 6).

**Fig. 10:**
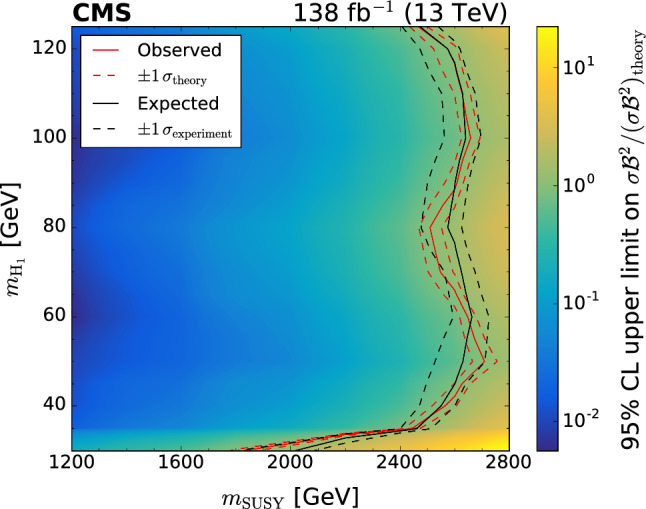
The observed 95% $\textrm{CL}$upper limit on $\sigma \mathcal {B} ^2 / (\sigma \mathcal {B} ^2)_{\text {theory}}$, quantified by the colour scale as a function of $m_{{\textrm{H}} _1}$ and $m_{\text {SUSY}}$. The solid and dashed red lines indicate the observed excluded region and its theoretical uncertainty, respectively. The solid and dashed black lines respectively represent the expected excluded region and its 68% $\textrm{CL}$ interval, under the background-only hypothesis

The $\sigma \mathcal {B} ^2$ upper limits are used in conjunction with the theoretical $\sigma$ and $\mathcal {B}$ values from Sect. 2 to exclude ranges of masses in $m_{{\textrm{H}} _1}$ and $m_{\text {SUSY}}$ in the benchmark model. The observed 95% $\textrm{CL}$upper limits on *r*, the ratio of measured and theoretical values of $\sigma \mathcal {B} ^2$, are shown in Fig. 10, with the corresponding exclusion contours at $r=1$. Masses $1200< m_{\text {SUSY}} < 2500\,\text {GeV}$ are excluded within the range $40< m_{{\textrm{H}} _1} < 120\,\text {GeV}$. Expected exclusion contours for the background-only scenario agree within one standard deviation with the observed contours. In the region $110< m_{{\textrm{H}} _1} < 125\,\text {GeV}$, $\mathcal {B}$ starts to decrease more quickly (as shown in Table 2), leading to a corresponding reduction in sensitivity. Most of the sensitivity at large $m_{\text {SUSY}}$ comes from the $H_{\textrm{T}}> 3500\,\text {GeV}$ region, where the statistical uncertainties in the observed yields are dominant over systematic uncertainties. This search does not explore the region outside of that shown in Fig. 10.

To aid reinterpretation of the search by reducing the model-dependence, limits evaluated using only the upper $H_{\textrm{T}}$category are presented in Appendix A. Tabulated results are provided in the HEPData record for this analysis [71].

## Summary

This paper presents a search for pairs of light Higgs bosons (${\textrm{H}} _1$) produced in supersymmetric cascade decays. The targeted final states have small amounts of missing transverse momentum and two  decays that are reconstructed as large-radius jets using substructure techniques. The search is based on data from $\hbox {pp}$ collisions collected by the CMS experiment at $\sqrt{s}=13\,\text {TeV}$ during 2016–2018, corresponding to an integrated luminosity of 138$\,\text {fb}^{-1}$.

With no evidence found for an excess of events beyond the background expectations of the standard model (SM), the results are interpreted in the next-to-minimal supersymmetric extension of the SM (NMSSM), where a “singlino” of small mass leads to squark and gluino cascade decays that can predominantly end in a highly Lorentz-boosted singlet-like ${\textrm{H}} _1$ and a singlino-like neutralino of small transverse momentum.

Upper limits are set on the product of the production cross section and the square of the  branching fraction of the ${\textrm{H}} _1$ for an NMSSM benchmark model with almost mass-degenerate gluinos and light-flavour squarks and branching fractions of unity for the cascade decays ending with the ${\textrm{H}} _1$. Under the assumption of an SM-like  branching fraction, ${\textrm{H}} _1$ bosons with masses in the range 40–120$\,\text {GeV}$ arising from the decays of squarks or gluinos with a mass of 1200 to 2500$\,\text {GeV}$ are excluded at 95% confidence level.

## Figures and Tables

**Fig. 11:**
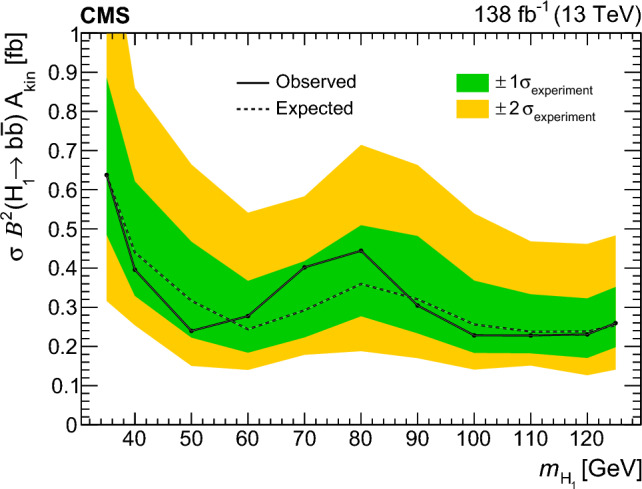
The observed and expected 95% $\textrm{CL}$ upper limit on the product of $\sigma \mathcal {B} ^2$ and $A_{\text {kin}}$, the kinematic acceptance and efficiency for the $H_{\textrm{T}}>3500\,\text {GeV}$ region, as a function of $m_{{\textrm{H}} _1}$. The results are independent of $m_{\text {SUSY}}$ within 10% in the range $1600<m_{\text {SUSY}} <2800\,\text {GeV}$. The solid and dashed black lines indicate the observed and median expected limits, respectively. The inner (green) band and the outer (yellow) band indicate the regions containing 68 and 95%, respectively, of the distribution of limits expected under the background-only hypothesis

## Data Availability

This manuscript has no associated data or the data will not be deposited. [Authors’ comment: Release and preservation of data used by the CMS Collaboration as the basis for publications is guided by the CMS policy as stated in https://cms-docdb.cern.ch/cgi-bin/PublicDocDB/RetrieveFile?docid=6032 &filename=CMSDataPolicyV1.2.pdf &version=2CMSdata preservation, re-use and open access policy.]

## A Simplified analysis for reinterpretation

To aid reinterpretation of the search, a simplified analysis is performed using only the 10 search regions in the upper $H_{\textrm{T}}$category. The value $A_{\text {kin}}$ is defined as the product of acceptance and efficiency for a signal event to satisfy the kinematic selection (defined in Sect. 5) and the $H_{\textrm{T}}>3500\,\text {GeV}$ requirement. The value of $A_{\text {kin}}$ is common among all 10 search regions in the simplified analysis, and is quoted for the benchmark signal model in Table 3. Upper limits on the product $\sigma \, \mathcal {B} ^2 \, A_{\text {kin}}$ as a function of $m_{{\textrm{H}} _1}$ are set in Fig. 11, from which $\sigma \mathcal {B} ^2$ limits for different signal models can be derived through division by the appropriate value of $A_{\text {kin}}$. Since the upper $H_{\textrm{T}}$category provides most of the sensitivity for $m_{\text {SUSY}} >2000\,\text {GeV}$ in the nominal analysis, the $\sigma \mathcal {B} ^2$ upper limits in this region are not much weaker in the simplified analysis. This is not the case in the region $m_{\text {SUSY}} <2000\,\text {GeV}$, where the lower $H_{\textrm{T}}$categories become important.

**Table 3:** Reference values of the product of kinematic acceptance and efficiency ($A_{\text {kin}}$) for the $H_{\textrm{T}}>3500\,\text {GeV}$ region for the benchmark signal model with different values of $m_{\text {SUSY}}$. These values are independent of $m_{{\textrm{H}} _1}$ within 2% in the range $30<m_{{\textrm{H}} _1} <125\,\text {GeV}$

$m_{\text {SUSY}}$ [$\text {GeV}$]	1600	2000	2200	2400	2600	2800
$A_{\text {kin}}$	0.17	0.46	0.58	0.66	0.71	0.74

The double-b  tag and mass region selections are not considered in $A_{\text {kin}}$. This is done for simplicity, and because the fraction of events satisfying these selections is not found to be strongly model-dependent (except for the dependence on $m_{{\textrm{H}} _1}$, which is accounted for explicitly in Fig. 11). For the benchmark model, this fraction is found to be independent of $m_{\text {SUSY}}$ within 10% in the region $1600<m_{\text {SUSY}} <2800\,\text {GeV}$ and $35<m_{{\textrm{H}} _1} <125\,\text {GeV}$. This approximate independence does not hold for models with $m_{\text {SUSY}} <1600\,\text {GeV}$, where the ${\textrm{H}} _1$ $p_{\textrm{T}}$ distribution has substantial contributions below the $p_{\textrm{T}}$ necessary for the  decay products to be merged in a single AK8 jet. Only models with typical  angular separation $\varDelta R<0.8$ should be considered for reinterpretation.

## References

[1] Ramond P (1971). Dual theory for free fermions. Phys. Rev. D.

[2] Y.A. Golfand, E.P. Likhtman, Extension of the algebra of Poincaré group generators and violation of P invariance. JETP Lett. **13**, 323 (1971). http://jetpletters.ru/ps/1584/article_24309.pdf

[3] Neveu A, Schwarz JH (1971). Factorizable dual model of pions. Nucl. Phys. B.

[4] D.V. Volkov, V.P. Akulov, Possible universal neutrino interaction. JETP Lett. **16**, 438 (1972). http://www.jetpletters.ru/ps/1766/article_26864.pdf

[5] Wess J, Zumino B (1974). A Lagrangian model invariant under supergauge transformations. Phys. Lett. B.

[6] Wess J, Zumino B (1974). Supergauge transformations in four dimensions. Nucl. Phys. B.

[7] Fayet P (1975). Supergauge invariant extension of the Higgs mechanism and a model for the electron and its neutrino. Nucl. Phys. B.

[8] Nilles HP (1984). Supersymmetry, supergravity and particle physics. Phys. Rep..

[9] Ellwanger U, Hugonie C, Teixeira AM (2010). The next-to-minimal supersymmetric standard model. Phys. Rep..

[10] Ellwanger U, Teixeira A (2014). NMSSM with a singlino LSP: possible challenges for searches for supersymmetry at the LHC. JHEP.

[11] Ellwanger U, Teixeira A (2015). Excessive Higgs pair production with little MET from squarks and gluinos in the NMSSM. JHEP.

[12] Titterton A (2018). Exploring sensitivity to NMSSM signatures with low missing transverse energy at the LHC. JHEP.

[13] CMS Collaboration, Search for physics beyond the standard model in events with high-momentum Higgs bosons and missing transverse momentum in proton-proton collisions at 13 TeV. Phys. Rev. Lett. **120**, 241801 (2018). 10.1103/PhysRevLett.120.241801. arXiv:1712.08501 10.1103/PhysRevLett.120.241801 29956995

[14] CMS Collaboration, Search for higgsinos decaying to two Higgs bosons and missing transverse momentum in proton-proton collisions at $$\sqrt{s}=13\,{\rm TeV}$$. JHEP **05**, 014 (2022). 10.1007/JHEP05(2022)014. arXiv:2201.04206

[15] ATLAS Collaboration, Search for pair production of higgsinos in final states with at least three $$b$$-tagged jets in $$\sqrt{s} = 13$$ TeV $$pp$$ collisions using the ATLAS detector. Phys. Rev. D **98**, 092002 (2018). 10.1103/PhysRevD.98.092002. arXiv:1806.04030

[16] CMS Collaboration, Searches for electroweak neutralino and chargino production in channels with Higgs, Z, and W bosons in pp collisions at 8 TeV. Phys. Rev. D **90**, 092007 (2014). 10.1103/PhysRevD.90.092007. arXiv:1409.3168

[17] CMS Collaboration, Search for higgsino pair production in $${\rm pp}$$ collisions at $$\sqrt{s}$$ = 13 TeV in final states with large missing transverse momentum and two Higgs bosons decaying via $${\rm H} \rightarrow {\rm b}\bar{{\rm b}}$$. Phys. Rev. D **97**, 032007 (2018). 10.1103/PhysRevD.97.032007. arXiv:1709.04896

[18] CMS Collaboration, Precision luminosity measurement in proton-proton collisions at $$\sqrt{s} =$$ 13 TeV in 2015 and 2016 at CMS. Eur. Phys. J. C **81**, 800 (2021). 10.1140/epjc/s10052-021-09538-2. arXiv:2104.01927 10.1140/epjc/s10052-021-09538-2 PMC8550658 34781320

[19] CMS Collaboration, CMS luminosity measurement for the 2017 data-taking period at $$\sqrt{s} = 13$$ TeV, CMS Physics Analysis Summary CMS-PAS-LUM-17-004 (2018). https://cds.cern.ch/record/2621960

[20] CMS Collaboration, CMS luminosity measurement for the 2018 data-taking period at $$\sqrt{s} = 13$$ TeV, CMS Physics Analysis Summary CMS-PAS-LUM-18-002 (2019). https://cds.cern.ch/record/2676164

[21] Beenakker W, Höpker R, Spira M, Zerwas PM (1997). Squark and gluino production at hadron colliders. Nucl. Phys. B.

[22] Kulesza A, Motyka L (2009). Threshold resummation for squark–antisquark and gluino-pair production at the LHC. Phys. Rev. Lett..

[23] Kulesza A, Motyka L (2009). Soft gluon resummation for the production of gluino–gluino and squark–antisquark pairs at the LHC. Phys. Rev. D.

[24] Beenakker W (2009). Soft-gluon resummation for squark and gluino hadroproduction. JHEP.

[25] Beenakker W (2011). Squark and gluino hadroproduction. Int. J. Mod. Phys. A.

[26] Beenakker W (2012). NNLL resummation for squark–antisquark pair production at the LHC. JHEP.

[27] Beenakker W (2013). Towards NNLL resummation: hard matching coefficients for squark and gluino hadroproduction. JHEP.

[28] Beenakker W (2014). NNLL resummation for squark and gluino production at the LHC. JHEP.

[29] Beenakker W (2016). NNLL-fast: predictions for coloured supersymmetric particle production at the LHC with threshold and Coulomb resummation. JHEP.

[30] Djouadi A, Kalinowski J, Spira M (1998). HDECAY: a program for Higgs boson decays in the standard model and its supersymmetric extension. Comput. Phys. Commun..

[31] Djouadi A, Kalinowski J, Mühlleitner M, Spira M (2019). HDECAY: twenty++ years after. Comput. Phys. Commun..

[32] CMS Collaboration, Performance of the CMS Level-1 trigger in proton–proton collisions at $$\sqrt{s} = 13$$ TeV. JINST **15**, P10017 (2020). 10.1088/1748-0221/15/10/P10017. arXiv:2006.10165

[33] CMS Collaboration, The CMS trigger system. JINST **12**, P01020 (2017). 10.1088/1748-0221/12/01/P01020. arXiv:1609.02366

[34] CMS Collaboration, The CMS experiment at the CERN LHC. JINST **3**, S08004 (2008). 10.1088/1748-0221/3/08/S08004

[35] Alwall J (2014). The automated computation of tree-level and next-to-leading order differential cross sections, and their matching to parton shower simulations. JHEP.

[36] Alwall J (2008). Comparative study of various algorithms for the merging of parton showers and matrix elements in hadronic collisions. Eur. Phys. J. C.

[37] Frixione S, Nason P, Ridolfi G (2007). A positive-weight next-to-leading-order Monte Carlo for heavy flavour hadroproduction. JHEP.

[38] Nason P (2004). A new method for combining NLO QCD with shower Monte Carlo algorithms. JHEP.

[39] Frixione S, Nason P, Oleari C (2007). Matching NLO QCD computations with parton shower simulations: the POWHEG method. JHEP.

[40] Alioli S, Nason P, Oleari C, Re E (2010). A general framework for implementing NLO calculations in shower Monte Carlo programs: the POWHEG BOX. JHEP.

[41] NNPDF Collaboration, Unbiased global determination of parton distributions and their uncertainties at NNLO and LO. Nucl. Phys. B **855**, 153 (2012). 10.1016/j.nuclphysb.2011.09.024. arXiv:1107.2652

[42] NNPDF Collaboration, Parton distributions for the LHC Run II. JHEP **04**, 040 (2015). 10.1007/JHEP04(2015)040. arXiv:1410.8849

[43] NNPDF Collaboration, Parton distributions with QED corrections. Nucl. Phys. B ** 877**, 290 (2013). 10.1016/j.nuclphysb.2013.10.010. arXiv:1308.0598

[44] NNPDF Collaboration, Parton distributions from high-precision collider data. Eur. Phys. J. C ** 77**, 663 (2017). 10.1140/epjc/s10052-017-5199-5. arXiv:1706.00428 10.1140/epjc/s10052-017-5199-5 PMC6956957 31997920

[45] Sjöstrand T (2015). An introduction to PYTHIA 8.2. Comput. Phys. Commun..

[46] CMS Collaboration, Event generator tunes obtained from underlying event and multiparton scattering measurements. Eur. Phys. J. C **76**, 155 (2016). 10.1140/epjc/s10052-016-3988-x. arXiv:1512.00815 10.1140/epjc/s10052-016-3988-x PMC4946872 27471433

[47] Skands P, Carrazza S, Rojo J (2013). Tuning PYTHIA 8.1: the Monash, tune. Eur. Phys. J. C.

[48] CMS Collaboration, Extraction and validation of a new set of CMS pythia8 tunes from underlying-event measurements. Eur. Phys. J. C **80**, 4 (2020). 10.1140/epjc/s10052-019-7499-4. arXiv:1903.12179 10.1140/epjc/s10052-019-7499-4 PMC6944267 31976986

[49] Czakon M, Mitov A (2014). TOP++: a program for the calculation of the top-pair cross-section at hadron colliders. Comput. Phys. Commun..

[50] Gavin R, Li Y, Petriello F, Quackenbush S (2011). FEWZ 2.0: a code for hadronic Z production at next-to-next-to-leading order. Comput. Phys. Commun..

[51] Gavin R, Li Y, Petriello F, Quackenbush S (2013). W physics at the LHC with FEWZ 2.1. Comput. Phys. Commun..

[52] Li Y, Petriello F (2012). Combining QCD and electroweak corrections to dilepton production in FEWZ. Phys. Rev. D.

[53] CMS Collaboration, Measurement of the inelastic proton–proton cross section at $$\sqrt{s}=13$$ TeV. JHEP **07**, 161 (2018). 10.1007/JHEP07(2018)161. arXiv:1802.02613

[54] GEANT4 Collaboration, GEANT4—a simulation toolkit. Nucl. Instrum. Meth. A **506**, 250 (2003). 10.1016/S0168-9002(03)01368-8

[55] CMS Collaboration, Particle-flow reconstruction and global event description with the CMS detector. JINST **12**, P10003 (2017). 10.1088/1748-0221/12/10/P10003. arXiv:1706.04965

[56] Cacciari M, Salam GP, Soyez G (2008). The anti-$$k_{\rm T }$$ jet clustering algorithm. JHEP.

[57] Cacciari M, Salam GP, Soyez G (2012). FastJet user manual. Eur. Phys. J. C.

[58] CMS Collaboration, Technical proposal for the Phase-II upgrade of the Compact Muon Solenoid, CMS Technical Proposal CERN-LHCC-2015-010, CMS-TDR-15-02 (2015). http://cds.cern.ch/record/2020886

[59] CMS Collaboration, Pileup mitigation at CMS in 13 TeV data. JINST **15**, P09018 (2020). 10.1088/1748-0221/15/09/p09018. arXiv:2003.00503

[60] Bertolini D, Harris P, Low M, Tran N (2014). Pileup per particle identification. JHEP.

[61] CMS Collaboration, Jet energy scale and resolution in the CMS experiment in pp collisions at 8 TeV. JINST **12**, P02014 (2017). 10.1088/1748-0221/12/02/P02014. arXiv:1607.03663

[62] CMS Collaboration, Jet energy scale and resolution performance with 13 TeV data collected by CMS in 2016, Detector Performance Report CMS-DP-2018-028 (2018). http://cds.cern.ch/record/2622157

[63] CMS Collaboration, Jet performance in pp collisions at $$\sqrt{s}=7$$ TeV, CMS Physics Analysis Summary CMS-PAS-JME-10-003 (2010). http://cdsweb.cern.ch/record/1279362

[64] CMS Collaboration, Jet algorithms performance in 13 TeV data, CMS Physics Analysis Summary CMS-PAS-JME-16-003 (2017). http://cds.cern.ch/record/2256875

[65] CMS Collaboration, Identification of heavy-flavour jets with the CMS detector in pp collisions at 13 TeV. JINST **13**, P05011 (2018). 10.1088/1748-0221/13/05/P05011. arXiv:1712.07158

[66] Adams T (2016). Beam test evaluation of electromagnetic calorimeter modules made from proton-damaged PbWO$$_4$$ crystals. JINST.

[67] Larkoski AJ, Marzani S, Soyez G, Thaler J (2014). Soft drop. JHEP.

[68] ATLAS and CMS Collaborations, Procedure for the LHC Higgs boson search combination in summer 2011, ATLAS/CMS joint note ATL-PHYS-PUB-2011-011, CMS-NOTE-2011-005 (2011). http://cds.cern.ch/record/1379837

[69] Junk T (1999). Confidence level computation for combining searches with small statistics. Nucl. Instrum. Meth. A.

[70] Read AL (2002). Presentation of search results: the $${{\rm CL}}_{\rm s}$$ technique. J. Phys. G.

[71] HEPData record for this analysis (2022). 10.17182/hepdata.114359

